# Potent Anti-Glioblastoma Effects of Next-Generation MNK Inhibitors

**DOI:** 10.3390/cancers18132086

**Published:** 2026-06-27

**Authors:** Candice Mazewski, Ricardo E. Perez, Purav P. Vagadia, Masha Kocherginsky, Gary E. Schiltz, Frank Eckerdt, Leonidas C. Platanias

**Affiliations:** 1 Robert H. Lurie Comprehensive Cancer Center of Northwestern University, Chicago, IL 60611, USA; 2 Division of Hematology-Oncology, Department of Medicine, Feinberg School of Medicine, Northwestern University, Chicago, IL 60611, USA; 3 Department of Chemistry, Northwestern University, Evanston, IL 60208, USA; 4 Department of Preventive Medicine-Biostatistics & Informatics, Feinberg School of Medicine, Northwestern University, Chicago, IL 60611, USA; 5 Department of Pharmacology, Feinberg School of Medicine, Northwestern University, Chicago, IL 60611, USA; 6 Department of Medicine, Jesse Brown Veterans Affairs Medical Center, Chicago, IL 60612, USA

**Keywords:** MAPK-interacting kinase (MNK), glioblastoma (GBM), DNA methyltransferase (DNMT), glioma stem cells (GSCs), apoptosis, invasion

## Abstract

**Background/Objectives**: Glioblastoma (GBM) remains one of the most aggressive and treatment-resistant malignancies, driven in part by heterogeneous, therapy-resistant glioma stem cells (GSCs). Improving clinical outcomes will require innovative therapeutic approaches that target unique molecular vulnerabilities. The mitogen-activated protein kinase (MAPK) pathway drives tumor progression across multiple cancers, including GBM. MAPK-interacting kinases (MNK1/2) represent MAPK downstream effectors that phosphorylate eukaryotic translation initiation factor 4E (eIF4E), a regulator of oncogenic and anti-apoptotic mRNA translation. We previously identified pharmacological MNK inhibition as a promising therapeutic strategy for GBM, but most available MNK inhibitors lack specificity. **Methods**: Novel MNK inhibitor compounds were developed using medicinal chemistry optimization and evaluated through molecular docking and kinome profiling analyses. Antineoplastic activity was assessed in established GBM cell lines and patient-derived glioma stem cell models cultured as 3-D neurospheres under stem cell-permissive conditions. Effects on MNK signaling, cell viability, neurosphere growth, migration, invasion, and apoptosis were analyzed using immunoblotting, flow cytometry, viability assays, wound healing assays, and 3-D invasion assays. In addition, a compound screen was performed to identify therapeutic agents that enhance MNK-targeted therapy, followed by validation using pharmacological inhibition and siRNA-mediated knockdown approaches. **Results**: Our next-generation MNK inhibitor NUCC-201893 exhibited high target specificity and greater potency than the lead compound NU808, effectively suppressing eIF4E phosphorylation, GBM cell viability, neurosphere growth, migration, and invasion. Compound screening identified DNA methyltransferase (DNMT) inhibition as a potent enhancer of MNK blockade. Pharmacological DNMT inhibition enhanced the cytotoxic effects of siRNA-mediated MNK1 knockdown, while concurrent pharmacological inhibition of MNKs and DNMT resulted in greater suppression of neurosphere growth and robust induction of apoptotic responses in GSCs. **Conclusions**: These findings identify dual MNK and DNMT inhibition as a promising combinatorial strategy that effectively triggers antineoplastic effects in GBM cells and GSCs.

## Simple Summary

Glioblastoma (GBM) is an aggressive brain cancer that is difficult to treat because many tumor cells develop resistance to therapy, particularly glioma stem cells (GSCs). In this study, we developed and characterized a new selective inhibitor of MAPK-interacting kinases (MNKs), termed NUCC-201893 (NU1893), which showed improved potency compared with our previous lead compound NU808. MNK inhibition reduced GBM cell viability, suppressed neurosphere growth, and inhibited invasive behavior in both established and patient-derived GBM models. Because MNK signaling can function as a survival pathway during therapeutic stress, we performed a drug screen to identify compounds that enhance MNK-targeted therapy and identified DNA methyltransferase (DNMT) inhibition as a potent combinatorial strategy. Combined MNK and DNMT inhibition strongly enhanced antitumor activity and induced apoptosis in therapy-resilient mesenchymal GSCs. These findings support selective MNK targeting, particularly in combination with pharmacological DNMT inhibition, as a promising therapeutic strategy for GBM.

## 1. Introduction

Treatment improvements of glioblastoma (GBM), the most aggressive form of brain cancer, continue to be elusive. Despite technological advances and novel target discoveries, the GBM median survival remains very low [1], the standard of care has not meaningfully improved in 20 years [2], and tumor recurrence is essentially inevitable [3]. Several key obstacles continue to limit therapeutic success in GBM, including intratumoral heterogeneity and the requirement for therapeutics to effectively cross the blood–brain barrier (BBB) [4,5,6]. Glioma stem cells (GSCs) are a critical subpopulation within this heterogeneous tumor ecosystem and play a key role in driving tumor recurrence and resistance to conventional GBM therapies [7,8,9,10]. In addition, the highly invasive nature of GBM cells, which infiltrate healthy brain tissue, represents a major concern of this highly malignant cancer [11,12]. Therefore, effective curative strategies for GBM should simultaneously address these obstacles to achieve meaningful improvements in clinical outcome.

Mitogen-activated protein kinase (MAPK) interacting kinases (MNKs) are important components of a chain of phosphorylation events leading to an impact on mRNA translation in GBM and in particular GSCs [13]. There are two of these serine/threonine kinases; MNK1 is mainly activated by stress and growth factors through MAPKs, p38 and ERK, while MNK2 has a high basal level of activity [14]. Research has shown that MNK1 and MNK2 are not required for regular cell growth and development, with knockout mice growing normally [14]. The most well-known function of MNKs is the phosphorylation and activation of eukaryotic initiation factor 4E (eIF4E), a key factor in cap-dependent mRNA translation; however, additional MNK downstream effectors include heterogeneous nuclear ribonucleoprotein A1 (hnRNPA1), Sprouty2, cytosolic phospholipase A2 (cPLA2), and pre-mRNA splicing factor (PSF), with lesser known roles [15]. Despite not being essential for regular cell growth and development, MNKs have been implicated in various cancers, most commonly in blood cancers [15]. MNK involvement in invasion was shown in a breast cancer model, where MNK1 knockout and a MNK inhibitor blocked progression to an invasive ductal carcinoma state [16]. Additionally, MNK relation to invasion has been seen in prostate cancer [17]. However, specific connections between MNKs and invasion in GBM have not yet been reported.

There has been some difficulty in developing MNK inhibitors that are specific and effective, and several previously described MNK inhibitors have shown limited clinical efficacy, which is why our group has taken on the task of developing potent and selective MNK inhibitors [18,19,20]. Increasing efforts have focused on combination strategies that target MNKs alongside complementary oncogenic pathways to enhance therapeutic efficacy of MNK inhibitors [21,22]. In particular, dual inhibition of MNKs and mTOR signaling has been extensively investigated, given their convergence on translational control and tumor cell survival [13,23,24,25,26,27]. These studies highlight the broader concept that MNK inhibition may be most effective when integrated into rational combination therapies that disrupt adaptive survival mechanisms.

In this context, epigenetic regulation has emerged as a critical contributor to tumor progression and therapeutic resistance. DNA methyltransferases (DNMTs) are key epigenetic modifiers that regulate gene expression through the addition of methyl groups to cytosine residues in CpG dinucleotides [28]. DNMT1 primarily functions in the maintenance of DNA methylation patterns during replication, whereas DNMT3A and DNMT3B mediate de novo methylation. Aberrant DNA methylation is a hallmark of cancer and is characterized by the simultaneous hypermethylation of tumor suppressor gene promoters and global hypomethylation, which can promote genomic instability and oncogene activation [28]. These epigenetic alterations contribute to tumor cell plasticity, immune evasion, and resistance to therapy, all of which represent features that are particularly relevant in GBM and the GSC population. Consequently, DNMT inhibitors have been actively explored as anticancer agents, with nucleoside analogs such as 5-azacytidine and decitabine already approved for the treatment of hematologic malignancies. In contrast, non-nucleoside DNMT inhibitors, which may offer improved specificity and reduced toxicity, remain under clinical development. Importantly, by reprogramming transcriptional states, DNMT inhibitors may disrupt adaptive resistance pathways and thereby sensitize tumor cells to other targeted therapies, providing a strong rationale for combinatorial approaches [29].

In the current study, we sought to determine the therapeutic potential of our newly developed MNK inhibitors in GBM. Specifically, we assessed their antiproliferative and anti-invasive effects. Our findings suggest potent antineoplastic effects of these inhibitors. Additionally, we found that pharmacological DNMT inhibition is an effective combination that enhances the antineoplastic capabilities of our MNK inhibitors, supporting a novel combinatorial strategy to more effectively target GBM and in particular, the therapy-resilient GSC subpopulation.

## 2. Materials and Methods

### 2.1. Synthesis of NU1893

The synthesis of this compound follows a route similar to that previously reported [18]. To a solution of 5-bromo-2,4-dichloropyrimidine (2.86 g, 12.55 mmol) in DMF (30 mL) was added 1H-benzo[d]imidazole (1.48 g, 12.55 mmol, 1 equiv) and DIPEA (2.43 g, 3.28 mL, 18.82 mmol, 1.5 equiv), and the reaction mixture was stirred for 16 h at room temperature, after which water was added (25 mL) until solids precipitated. The mixture was stirred for 5 min, then filtered to give 1-(5-bromo-2-chloropyrimidin-4-yl)-1H-benzo[d]imidazole (2.2 g, 57%) as a white solid. ^1^H NMR (500 MHz, CDCl3) δ 8.91 (s, 1H), 8.77 (s, 1H), 7.94–7.83 (m, 2H), 7.51–7.37 (m, 2H).

To a mixture of 1-(5-bromo-2-chloropyrimidin-4-yl)-1H-benzo[d]imidazole (700.0 mg, 2.26 mmol) and tert-butyl ((1s,3s)-3-hydroxycyclobutyl)carbamate (423.4 mg, 2.26 mmol, 1 equiv) was added DMF (7 mL), after which the mixture was immersed in an ice/water bath and subjected to an atmosphere of nitrogen. Sodium hydride (108.5 mg, 60% wt, 2.71 mmol, 1.2 equiv) was added, and the mixture was stirred for 1 h, after which the reaction was quenched with water, stirred vigorously for 2 min, then filtered to isolate tert-butyl((1s,3s)-3-((2-(1H-benzo[d]imidazol-1-yl)-5-bromopyrimidin-4-yl)oxy)cyclobutyl)carbamate (1.04 g, quant.) as a white solid, which was used without further purification.

To a mixture of tert-butyl ((1s,3s)-3-((2-(1H-benzo[d]imidazol-1-yl)-5-bromopyrimidin-4-yl)oxy)cyclobutyl)carbamate (1.04 g, 2.26 mmol) in dichloromethane (5 mL) was added TFA (5.33 mL, 69.21 mmol, 30 equiv), and the reaction was stirred for 16 h at room temperature, after which the mixture was concentrated to an oil, which was triturated from diethyl ether, stirred and filtered to isolate solids. The filtrate was discarded. Given the low solubility of this compound in dichloromethane, the collected solids were purified further by trituration by stirring in dichloromethane at elevated temperature (35 °C), then filtering to isolate (1s,3s)-3-((2-(1H-benzo[d]imidazol-1-yl)-5-bromopyrimidin-4-yl)oxy)cyclobutan-1-amine, Trifluoracetate (731 mg, 68.04%). ^1^H NMR (500 MHz, CD3OD) δ 9.17 (s, 1H), 8.78 (s, 1H), 8.59–8.53 (m, 1H), 7.81–7.75 (m, 1H), 7.50 (ddd, *J* = 8.4, 7.3, 1.3 Hz, 1H), 7.44 (ddd, *J* = 8.4, 7.3, 1.2 Hz, 1H), 5.42 (p, *J* = 7.2 Hz, 1H), 3.78–3.68 (m, 1H), 3.17 (dtt, *J* = 9.3, 7.1, 2.5 Hz, 2H), 2.51 (dddd, *J* = 10.4, 8.6, 7.4, 3.0 Hz, 2H). ^13^C NMR (126 MHz, CD3OD) δ 165.00, 159.83, 153.85, 143.13, 141.90, 131.23, 125.01, 124.20, 119.02, 115.40, 101.87, 66.09, 38.16, 35.37.

Proton (^1^H), and carbon (^13^C) NMR spectra were recorded on a 500 MHz Bruker Avance III spectrometer with a direct cryoprobe. Chemical shifts were reported in parts per million (δ) and were referenced using residual nondeuterated solvent as an internal standard (CDCl_3_ at 7.24 ppm for ^1^H NMR and 77.0 for ^13^C NMR; CD_3_OD at 3.33 ppm for ^1^H NMR and 47.6 for ^13^C NMR). NMR spectra are available in the Supplement Figure S6.

### 2.2. Cellular Flow Cytometric eIF4E Phosphorylation Assay

Supersignal eIF4E phosphorylation assay was performed using LN229 cells, as previously described [19].

### 2.3. Cell Culture and Reagents

LN229 (cat. # CRL-2611; RRID: CVCL_0393) and U87 (cat. # HTB-14; RRID: CVCL_0022) cells were obtained from American Type Culture Collection (Manassas, VA, USA) and maintained as 2-D cultures in DMEM with 10% FBS and 1% gentamycin at 37 °C in 5% CO_2_. The patient-derived xenograft (PDX) line GBM43 and the glioma stem cell (GSC) line 83Mes are well established in our laboratory [13,30]. GBM43, 83Mes, and for some experiments LN229 cells, were grown as 3-D neurosphere cultures in cancer stem cell (CSC) media (DMEM/F12, B27 supplement (2%), heparin (5 µg/mL), EGF (20 ng/mL), FGF (20 ng/mL), and gentamycin (1 mg/mL), as described [30]. Cells were tested every 6 months by short tandem repeat (STR) analysis and authenticated according to published STR profiles, where available. Cell lines were regularly tested for *Mycoplasma*. SGI-1027 (#HY-13962) was purchased from MedChemExpress (Monmouth Junction, NJ, USA). A list of reagents and tools can be found in Key Resources Table (Table S1).

### 2.4. Cell Viability Assay

The cell viability assay was previously described [25]. In brief, in a 96-well plate, 2000 cells were seeded per well. Treatments were applied with DMSO as vehicle control (VC). Five days after treatment, WST-1 Cell Proliferation Assay (Roche) reagent (10% *v*/*v*) was added, and the plate absorbance was read at 440 nm after 1 h and up to 4 h. Viability was determined based on the absorbance, as a percentage of the absorbance of the vehicle control cells. GraphPad Prism v.10 was used to calculate IC_50_ values.

### 2.5. Neurosphere Assay

Neurospheres were analyzed using the previously developed acridine orange methodology from our group [31]. Briefly, in ultra-low-attachment 96-well round-bottom Corning (Corning, NY, USA) plates, 500–1000 cells were seeded per well in CSC media and gently spun down to form a single sphere at the bottom of each well. Inhibitors or VC were added the next day, and spheroids were gently spun down. Seven days later, 10 µL of acridine orange (1 µg/mL) was added to each well and incubated at 37 °C for at least 1 h. Plates were read on a Cytation 3 Biotek (Winooski, VT, USA) microplate reader. The cross-sectional area was determined for each spheroid, and the cross-sectional areas of the treatment spheres were compared to vehicle control spheres using Cytation 3 software Gen5 v 2.09.

### 2.6. Wound Healing Assay

LN229 cells were seeded at 500,000 per well in a 6-well plate. The next day, the media were replaced with 0.5% FBS DMEM. The following day, a scratch was made with a 200 µL pipette tip in the center of each well. Wells were washed with PBS twice, and then 0.5% FBS-containing media were added back, with the appropriate treatments: MNK inhibitors or vehicle control (VC). Pictures of the scratch were taken at 0 h and then 24 h later. ImageJ 1.53k and the “Wound healing size tool” plugin were used to assess the area devoid of cells.

### 2.7. 3-D Invasion Assay

The Cultrex 3-D Spheroid BME Cell Invasion Assay (R&D Systems, Minneapolis, MN, USA) was described previously [32]. Briefly, 3000 cells were seeded per well in Costar 96-Well Round-Bottom Ultra-Low Attachment plates as single cell suspension in cancer stem cell media and 1× spheroid formation ECM. After two days and confirmation that spheres were formed, the invasion matrix was added, and the treatments were added an hour later. Pictures were taken on the day of treatment and then on days 3, 4, 5, 6, and 7. Quantitative analysis of invaded area (minus the inner sphere) was done as described [32].

### 2.8. Cell Lysis and Immunoblotting

Cells were treated for 90 min with the indicated MNK inhibitors compared to a DMSO vehicle control for phosphorylation of eIF4E reduction analysis. For apoptosis markers, GSCs were treated for 72 h with MNK inhibitors, SGI-1027, or the combination. For all other immunoblots, cells were treated for 4 h. Cell lysis was done in lysis buffer (50 mM Tris pH 7.5, 150 mM NaCl, 5 mM EDTA, 0.5% NP40, supplemented with protease and phosphatase inhibitors), followed by SDS-PAGE and immunoblotting. Antibodies against phosphorylated eIF4E at S209 (#9741), PARP (#9542), cleaved caspase 3 (#9661) BAX (#2772), and MNK1 (#2195) were purchased from Cell Signaling Technology. Antibodies against eIF4E (#sc-9976), HSP-90 (#sc-7947) were purchased from Santa Cruz Biotechnology (Dallas, TX, USA). A BioRad ChemiDoc Imager (Bio-Rad Laboratories, Hercules, CA, USA) was used to detect the chemiluminescence of the protein bands and ImageLab 6.1 software (Bio-Rad Laboratories, Hercules, CA, USA) was used to obtain blot pictures.

### 2.9. RNA Isolation and Real-Time Quantitative PCR

Total RNA was isolated using RNeasy Mini Kit (QIAGEN, Germantown, MD, USA); 2 µg of total RNA was reverse transcribed into cDNA using the High Capacity cDNA Reverse Transcription Kit (Applied Biosystems, Waltham, MA, USA). *GAPDH* was used as a normalization control. Quantitative PCR reactions were performed on a QuantStudio 6 Flex real-time PCR system (ThermoFisher, Waltham, MA, USA) with the following conditions: activation at 50 °C; 2 min and 95 °C; 20 s, 50 cycles of denaturation at 95 °C; 1 s and annealing/extension at 60 °C; 20 s. TaqMan Fast Advanced Master mix (ThermoFisher) was used to carry out quantitative PCR reactions. Relative gene expression was analyzed by the ^ΔΔ^C_t_ method.

### 2.10. Annexin V Apoptosis Assay

For analysis of apoptosis, the BD Pharmingen FITC Annexin V Apoptosis Detection Kit I (BD Biosciences, Milpitas, CA, USA) was used. Briefly, 83Mes cells grown in CSC medium under stem cell-permissive conditions were seeded in a 6-well plate, and treatment was added. After 72 h, cells were collected and stained with Annexin V FITC and propidium iodide using the manufacturer’s instructions. Flow was analyzed on 10,000 cells using an LSRFortessa instrument (BD Biosciences). FlowJo v10.6 software (Tree Star) was used for gating, quadrant quantification, and generating associated dot plots.

### 2.11. MKNK1 and MKNK2 siRNA Knockdowns

Seven hundred thousand LN229 cells were seeded in 10 cm dishes. The next day, RNAiMAX (ThermoFisher) and ON-TARGETplus SMARTpool non-targeting control, *MKNK1*-, or *MKNK2*-targeting siRNAs (Horizon, Lafayette, CO, USA) were added to Opti-Mem media separately and combined after 5 min. After 30 min, the mixture was added dropwise to the cells. The next day, the media were aspirated, and fresh media were added. The following day, cells were collected for immunoblotting and RNA isolation or seeded for viability and neurosphere assays.

### 2.12. Kinome Screen

A kinome screen for compound NUCC-201893 was performed by Eurofins DiscoverX Corporation (San Diego, CA, USA) (Item: 87-0002-1000) using the KINOMEscan™ screening platform and testing against a panel of 97 kinases. The methodological details were provided by the company as described below. Kinase-tagged T7 phage strains were grown in parallel in 24-well blocks in an *E. coli* host derived from the BL21 strain. *E. coli* were grown to log-phase and infected with T7 phage from a frozen stock (multiplicity of infection = 0.4) and incubated with shaking at 32 °C until lysis (90–150 min). The lysates were centrifuged (6000× *g*) and filtered (0.2 μm) to remove cell debris. The remaining kinases were produced in HEK-293 cells and subsequently tagged with DNA for qPCR detection. Streptavidin-coated magnetic beads were treated with biotinylated small molecule ligands for 30 min at room temperature to generate affinity resins for kinase assays. The liganded beads were blocked with excess biotin and washed with blocking buffer (SeaBlock (Pierce), 1% BSA, 0.05% Tween 20, 1 mM DTT) to remove unbound ligand and to reduce non-specific phage binding. Binding reactions were assembled by combining kinases, liganded affinity beads, and test compounds in 1× binding buffer (20% SeaBlock, 0.17× PBS, 0.05% Tween 20, 6 mM DTT). Test compounds were prepared as a 100× stock in 100% DMSO and directly diluted into the assay. All reactions were performed in polypropylene 384-well plates in a final volume of 0.02 mL. The assay plates were incubated at room temperature with shaking for 1 h and the affinity beads were washed with wash buffer (1× PBS, 0.05% Tween 20). The beads were then re-suspended in elution buffer (1× PBS, 0.05% Tween 20, 0.5 μM non-biotinylated affinity ligand) and incubated at room temperature with shaking for 30 min. The kinase concentration in the eluates was measured by qPCR.

### 2.13. Statistics

All statistical analyses were performed using GraphPad Prism v. 10. Sample sizes, number of replicates, and the appropriate statistical tests used are described in detail in each figure legend.

## 3. Results

### 3.1. Design and Synthesis of New MNK Inhibitor NUCC-201893 (NU1893)

**Figure 1:**
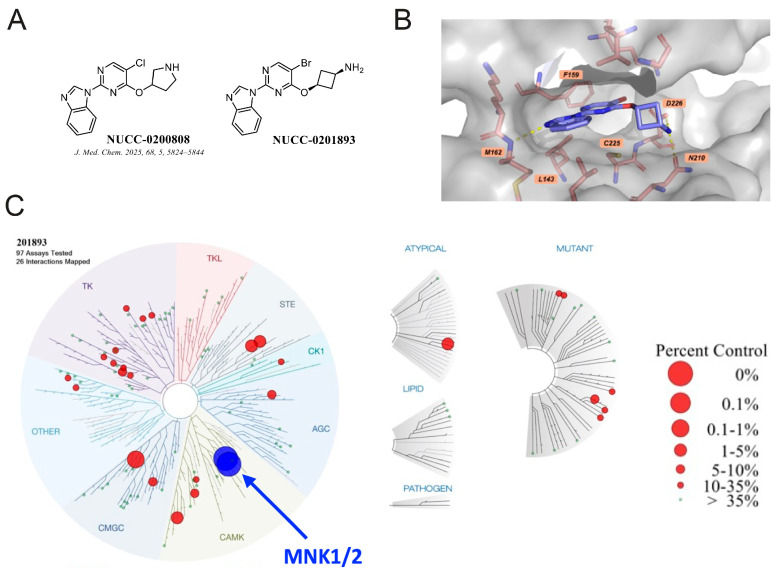
Characterization of novel MNK inhibitor NU1893. (**A**) Structures of previously reported MNK inhibitor NU808 (**left**) and the new compound NU1893 (**right**) [18]. (**B**) Structure of inhibitor NU1893 docked into MNK2-D228G. (**C**) Kinome plot depicting the kinase selectivity of NU1893 across 97 kinases (10 μM concentration). Spot cutoff value: 35% of control. MNK1 and MNK2 are both highlighted in blue.

In recent work, we described the development of NUCC-200808 (NU808) [18] (Figure 1A, left panel) which contains a chlorine in the C-5 position of the pyrimidine and an ether moiety with a secondary amine on the right-hand side. In our published crystal structure of the single enantiomer of NUCC-0200808 (NUCC-0201049) bound to MNK2-D228G (PDB entry 9HRC), we observed that Leu-143 and Phe-159 residues were adjacent to the C-5 chlorine on the pyrimidine ring. In efforts to intensify the hydrophobic interaction between the halogen and Phe-159 residue, we introduced a bromine atom in the C-5 position. In our published crystal structure [18], we also observed that an Asp-226 residue was adjacent to the pyrrolidine nitrogen on the right hand side of our compound. To modify the positioning of this amine and increase the hydrogen bonding interaction, we introduced a primary amine functionality. The resulting compound, NUCC-201893 (NU1893) (Figure 1A, right panel), was synthesized via a similar route as that used to synthesize NU808 in our previous work [18].

### 3.2. Structure and Activity of NUCC-201893

To evaluate the binding mode of our inhibitor NUCC-0201893 (NU1893), we docked NU1893 into the MNK2-D228G structure (Figure 1B). We extracted the ligand from our previously reported crystal structure (PDB 9HRC) and docked NU1893 into the binding pocket using Boltz-2. The overall binding mode was predicted to be essentially identical as our previous ligand NUCC-0201049 [18]. Key interactions we observed were a hydrogen bond between the benzimidazole and the Met-162 hinge residue. Notably, we also observed a hydrogen bond between the cyclobutyl amine and both D226 and N210, which is consistent with improved inhibition. Changing the chlorine to a bromine on the pyrimidine ring also appeared to more fully fill the open back pocket of the binding site.

To evaluate the kinase selectivity of NU1893, we performed kinome profiling with a diverse panel of 97 kinases. This profiling revealed that compound NU1893 displayed an overall binding profile similar to our previously developed MNK inhibitor NU808 [18], although it engaged a broader set of kinases. NU1893 was found to interact with a total of 23 kinases at a cutoff value of 35% of control, two of which were MNK1 and MNK2, which showed the strongest binding (Figure 1C and Table S2). Whereas NU808 showed a lower affinity for MNK1 relative to MNK2, NU1893 exhibited the opposite trend, displaying a stronger preference for MNK1 compared to MNK2 (Table S2). These results indicate that NU1893 is a specific MNK inhibitor with slightly higher selectivity for MNK1 over MNK2.

### 3.3. NU808 and NU1893 Inhibit Downstream Phosphorylation of eIF4E and Reduce Viability of Glioblastoma Cells

**Figure 2:**
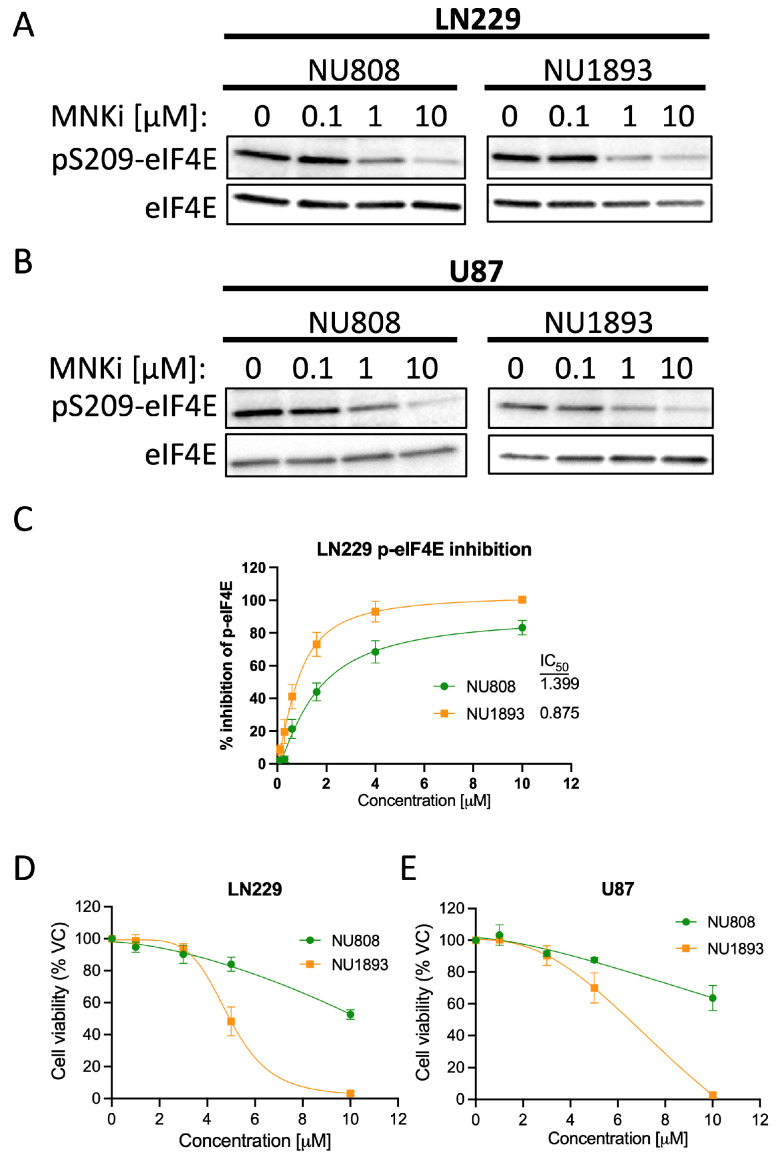
Effects of MNK-targeting compounds on MNK signaling and cell viability. (**A**,**B**) LN229 (**A**) or U87 (**B**) cells were treated with DMSO as vehicle control (VC) or increasing concentrations of MNK inhibitor compounds NU808 (**left**) and NU1893 (**right**) for 90 min and then processed for SDS-PAGE and immunoblotting with indicated antibodies. (**C**) LN229 cells were treated with indicated compounds for 3 h at 0.1, 0.3, 0.6, 1.6, 4.0, and 10.0 μM and subjected to cellular flow cytometric eIF4E phosphorylation assay using an Alexa 647 conjugated phospho-Ser-209 specific eIF4E antibody. GraphPad Prism v.10 was used to determine IC_50_ values. Data represent means ± SEM of 4 independent experiments. (**D**,**E**) LN229 (**D**) or U87 (**E**) cells were treated with indicated compounds at increasing concentrations for 5 days, and cell viability was determined using the cell proliferation reagent, WST-1. Data represent means ± SEM of 4 (LN229) or 3 (U87) independent experiments, each done in triplicate.

Next, we evaluated both compounds in cellular assays to determine their ability to inhibit phosphorylation of eIF4E at Ser-209 (pS209), a well-characterized MNK1/2 downstream target, in brain cancer cells [13,25,26]. Treatment with 1 µM of either NU808 and NU1893 markedly reduced pS209 levels in LN229 and U87 cells, confirming potent inhibition of MNK activity (Figure 2A,B and Figure S1). Quantification of pS209 by flow cytometric eIF4E phosphorylation assay [19] in LN229 cells revealed IC_50_ values of 1.597 µM for NU808 and 0.886 µM for NU1893, indicating that both compounds effectively target catalytic MNK activity as judged by phosphorylation of eIF4E on Ser-209, with NU1893 exhibiting superior potency over NU808 in cellular assays (Figure 2C).

We next asked whether inhibition of eIF4E phosphorylation translated into reduced cell viability. While NU808 exhibited only modest effects, NU1893 decreased viability of both LN229 and U87 cells grown under standard 2-D culture conditions (Figure 2D,E). Together, the dose-dependent reduction of cell viability largely validates the kinase inhibitory capacity of our compounds, with NU1893 exhibiting strongly increased potency over NU808.

### 3.4. Compound Efficacy in 3-D Glioma Stem Cell Models

**Figure 3:**
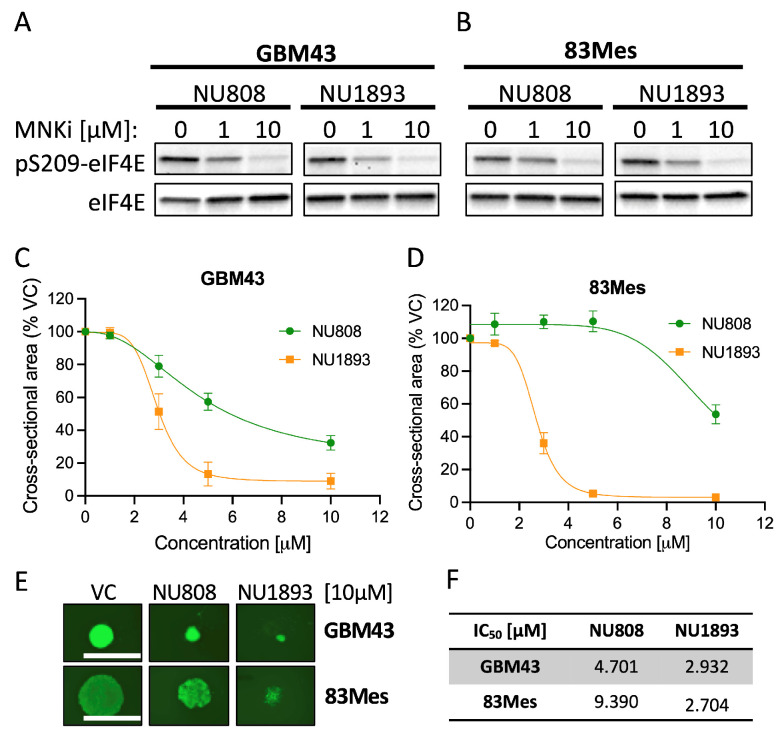
Effects of MNK-targeting compounds on MNK signaling and 3-D growth as multipotent neurosphere cultures. (**A**,**B**) The PDX line GBM43 (**A**) or the GSC line 83Mes (**B**) were propagated as neurospheres under stem cell-permissive conditions and treated with DMSO, 1 μM, or 10 μM of MNK inhibitor compounds NU808 (**left**) or NU1893 (**right**) for 90 min and then processed for SDS-PAGE and immunoblotting with indicated antibodies. (**C**,**D**) GBM43 (**C**) or 83Mes (**D**) spheroids were propagated in serum-free CSC medium in round-bottom 96-well plates in the presence of DMSO (VC) or the indicated drugs at increasing concentrations. After 7 days, spheres were stained with acridine orange and imaged to determine cross-sectional area. Data represent means ± SEM of 3 independent experiments, each done in duplicate. (**E**) Representative images of neurospheres treated with 10 μM of indicated MNK inhibitor compounds as shown in (**C**,**D**). Scale bar, 1000 μm. (**F**) IC_50_ values of experiments in (**C**,**D**), as determined by GraphPad Prism v.10.

Three-dimensional (3-D) spheroid cultures are generally recognized as more physiologically relevant than conventional 2-D monolayer cultures for modeling glioma biology and therapeutic response [30,33,34]. In addition, patient-derived lines propagated in serum-free, stem cell-permissive media form multipotent neurospheres enriched for glioma stem-like cells with enhanced self-renewal capacity [35,36]. To evaluate the effects of our MNK inhibitor compounds in such systems, we employed the patient-derived xenograft (PDX) line GBM43 [13], and the established glioma stem cell line 83Mes [25,30], grown as 3-D neurospheres under stem cell-permissive conditions. Both compounds effectively reduced phosphorylation of eIF4E at Ser-209 in GBM43 (Figure 3A and Figure S2) and 83Mes (Figure 3B and Figure S2) 3-D cultures, confirming on-target inhibition of MNK signaling also in these 3-D systems. This biochemical inhibition was associated with marked suppression of neurosphere growth in GBM43 (Figure 3C) and 83Mes (Figure 3D) spheroids. However, across all 3-D models tested, NU1893 consistently produced stronger inhibition of MNK downstream signaling (Figure 3A,B) and greater suppression of neurosphere growth (Figure 3C–F) than NU808, validating its superior potency also in glioma stem-like context.

### 3.5. MNK Inhibitors Reduce Migration of LN229 Cells

**Figure 4:**
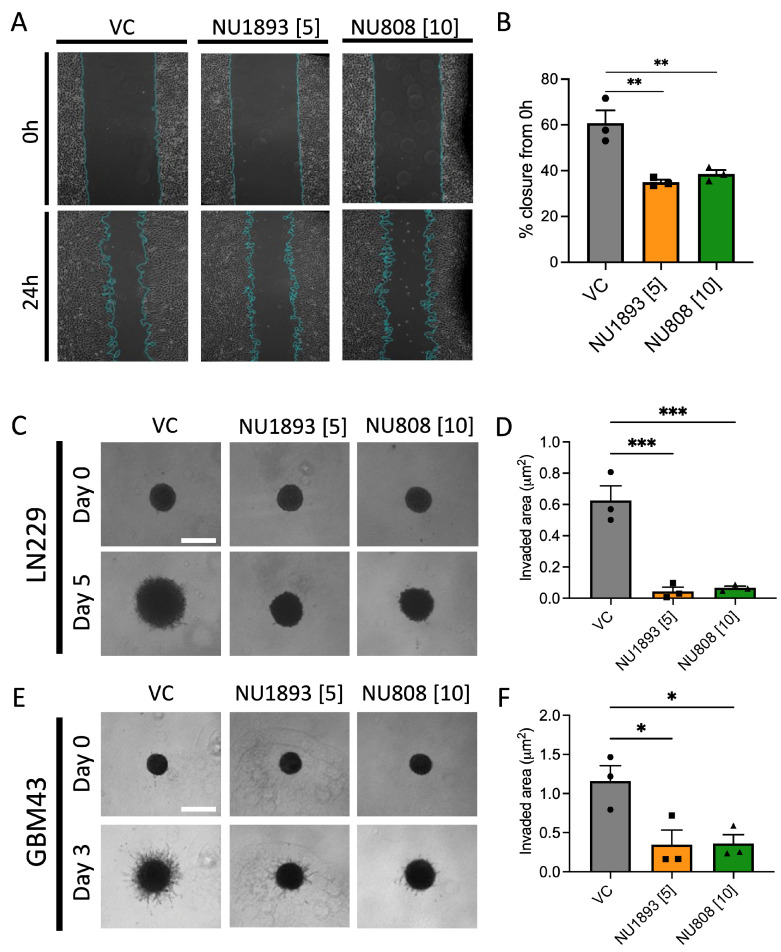
Effects of MNK inhibition on 2-D migration and 3-D invasion. (**A**) Representative images of LN229 cells subjected to the wound healing (scratch) assay with NU808 at 10 μM or NU1893 at 5 μM. (**B**) Quantification of experiments as shown in (**A**). Data represent means ± SEM of 3 independent experiments. Statistical significance comparing individual treatment groups to the vehicle control (VC) group was assessed using an ordinary one-way analysis of variance (ANOVA) followed by Dunnett’s multiple comparison test. **, *p* < 0.01. (**C**–**F**) LN229 (**C**,**D**) or GBM43 (**E**,**F**) grown in CSC medium as neurospheres were subjected to 3-D Matrigel invasion assay in the presence of DMSO (VC), NU808 (10 μM), or NU1893 (5 μM). (**C**,**E**) Representative images of invasion spheres for LN229 (**C**) and GBM43 (**E**). Scale bar, 500 μm. (**D**,**F**) Quantification of 3-D invasion assays as shown in (**C**,**E**). Data are expressed as invaded area by each spheroid, and bar graphs represent means ± SEM of 3 spheroids. Statistical significance comparing individual treatment groups to the control (VC) group was assessed using an ordinary one-way ANOVA followed by Dunnett’s multiple comparison test. *, *p* < 0.05; ***, *p* < 0.001.

Migration and invasion of GBM cells into surrounding healthy tissue contributes to the aggressiveness of the disease [37]. When subjected to wound healing assays, both compounds potently inhibited gap closure of LN229 cells with NU1893 at 5 μM showing potencies comparable to NU808 at 10 μM (Figure 4A,B). Thus, similar to our previous observations in conventional 2-D cell biological assays (Figure 2), NU1893 appears to exhibit superior efficacy over NU808 also in the context of GBM cell migration, as assessed by wound healing assays.

### 3.6. MNK Inhibitor Compounds Block GBM Invasion in 3-D

Infiltrative invasion of GBM cells into surrounding brain parenchyma complicates surgical resection and contributes to GBM aggressiveness, recurrence, and therapeutic resistance [3,37]. To investigate the role of MNKs in GBM invasiveness, we subjected cells to 3-D Matrigel invasion assays under stem cell-permissive culture conditions. Both LN229 (Figure 4C,D) and GBM43 (Figure 4E,F) neurospheres showed potent invasive properties and treatment with either compound substantially suppressed invasion (Figure 4C–F). Again, consistent with our previous observations in conventional 2-D assays (Figure 2 and Figure 4A,B), NU1893 also achieved comparable anti-invasive effects at half the concentration of NU808 in 3-D invasion assays. Together, these results demonstrate that pharmacological inhibition of MNKs by our compounds efficiently inhibits the invasive potential of GBM neurospheres, with NU1893 exhibiting greater potency than NU808.

### 3.7. Pharmacological Targeting of DNMT Increases Vulnerability to MNK Inhibition in GBM

**Figure 5:**
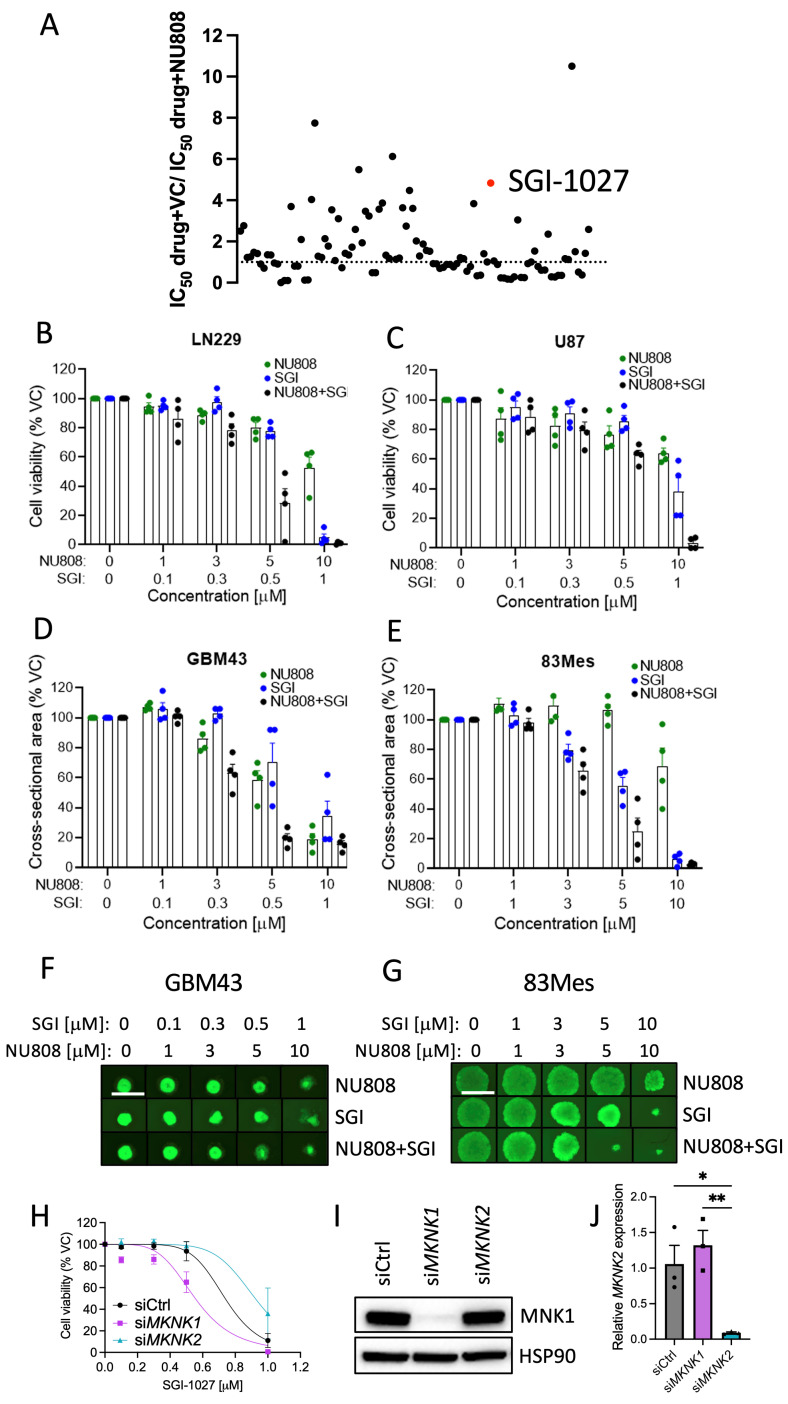
Effects of the DNMT inhibitor SGI-1027 in combination with MNK inhibitor NU808. (**A**) The top 104 hits from the compound screen in LN229 viability assays using NU808, or DMSO as control, in increasing concentrations (6 doses). Depicted are points representing individual compounds. Red point represents the DNMT inhibitor SGI-1027. (**B**,**C**) LN229 (**B**) or U87 (**C**) cells were treated with increasing concentrations of compound NU808 (1 to 10 μM) and/or SGI-1027 (0.1 to 1 μM) for 5 days, and cell viability was determined using the cell proliferation reagent, WST-1. (**D**,**E**) GBM43 (**D**) or 83Mes (**E**) spheroids were propagated in serum-free CSC medium in round-bottom 96-well plates in the presence of NU808 (1 to 10 μM) and/or SGI-1027 (0.1 to 1 μM for GBM43 or 1 to 10 μM for 83Mes). After 7 days, spheres were stained with acridine orange and imaged to determine cross-sectional area. (**B**–**E**) Data represent means ± SEM of 4 independent experiments, each done in duplicate. (**F**,**G**) Representative images of neurospheres from experiments shown in (**D**,**E**). Scale bar, 1000 μm. (**H**) LN229 cells were transfected with either control siRNA or siRNAs targeting *MKNK1* or *MKNK2*. The next day, cells were seeded into 96-well plates and treated with DMSO (VC) or increasing concentrations of SGI-1027 (0.1 to 1 μM) for 5 days, and cell viability was determined using the cell proliferation reagent, WST-1. Data represent means ± SEM of 3 independent experiments, each done in triplicate. (**I**) Immunoblot analysis of siRNA transfected cells from experiment in (**H**) using the indicated antibodies. (**J**) Gene expression analysis of siRNA transfected cells from experiment in (**H**). mRNA expression of *MKNK2* was assessed by quantitative RT-PCR using *GAPDH* for normalization. Data are expressed as fold-change relative mRNA expression of siCtrl-transfected LN229 cells and represent means ± SEM of three independent experiments, each done in triplicate. Statistical significance was assessed using an ordinary one-way ANOVA followed by Tukey’s multiple comparison test. *, *p* < 0.05; **, *p* < 0.01.

Our previous work indicated that MNK inhibition in brain cancer may require combination therapy due to the activation of compensatory signaling pathways or feedback loops [25,26]. To identify agents that enhance the anti-GBM activity of our lead MNK inhibitor NU808, we conducted a compound screen using a panel of approved cancer drugs (Table S3). Based on LN229 viability assays, we identified SGI-1027, a lipophilic, quinoline-based, non-nucleoside DNA methyltransferase (DNMT) inhibitor [38], as one of the top hits (Figure 5A and Table S3). Dose response validation confirmed that SGI-1027 potentiated the antineoplastic effects of NU808, substantially reducing cell viability in LN229 (Figure 5B) and U87 (Figure 5C) cells, as well as neurosphere growth in patient-derived GBM43 (Figure 5D) and 83Mes (Figure 5E) models. Notably, LN229, U87, and GBM43 models were responsive to lower concentrations of SGI-1027 (Figure 5B–D,F), while 83Mes spheroids required higher SGI-1027 concentrations (Figure 5E,G). This therapeutic resilience is in line with the notion of 83Mes representing the highly resistant subpopulation of mesenchymal GSCs [8,25].

To delineate the relative contributions of MNK1 and MNK2 to the antiproliferative effects observed after dual MNK and DNMT inhibition, we performed siRNA-mediated knockdown of each kinase in LN229 cells. Upon SGI-1027 treatment, MNK1 depleted cells exhibited markedly enhanced reduction in viability, whereas MNK2 knockdown lacked such effects (Figure 5H). Immunoblot analysis confirmed efficient depletion of MNK1 (Figure 5I and Figure S3), while MNK2 knockdown efficiency was verified at the transcript level due to the lack of a reliable antibody (Figure 5J). Together, these results indicate that SGI-1027 synergizes predominantly with inhibition of MNK1 rather than MNK2 in LN229 cells.

**Figure 6:**
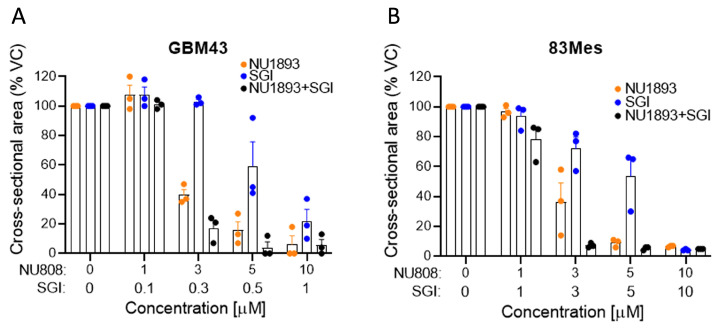
Effects of compound NU1893 in combination with SGI-1027. (**A**,**B**) GBM43 (**A**) or 83Mes (**B**) spheroids were propagated in serum-free CSC medium in round-bottom 96-well plates in the presence of DMSO (VC) or NU1893 (1 to 10 μM) and/or SGI-1027 (0.1 to 1 μM for GBM43 or 1 to 10 μM for 83Mes). After 7 days, the spheres were stained with acridine orange and imaged to determine cross-sectional area. Data represent means ± SEM of 4 independent experiments, each done in duplicate.

Next, we tested whether SGI-1027 also increased sensitivity to our next-generation MNK inhibitor compound NU1893 and confirmed that also NU1893 exhibited potent potential in combination with SGI-1027 in GBM43 (Figure 6A) and 83Mes (Figure 6B) neurospheres.

### 3.8. Combined DNMT and MNK Inhibition Is Required for Robust Induction of Apoptosis

**Figure 7:**
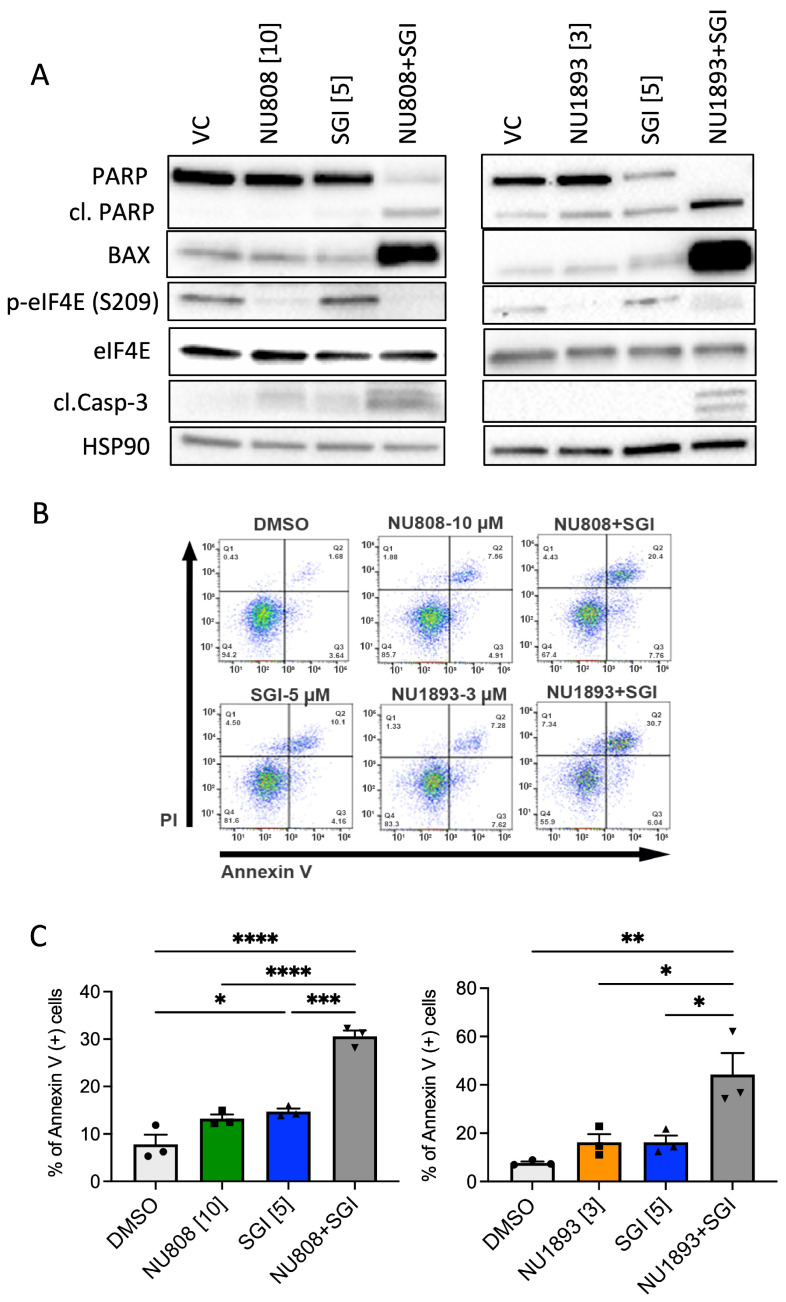
Proapoptotic effects of MNK inhibition in combination with SGI-1027 in mesenchymal GSCs. (**A**) 83Mes spheroids were treated with SGI-1027 at 5 μM in combination with either the MNK inhibitor NU808 at 10 μM (**left**) or NU1893 at 3 μM (**right**) for 72 h and then processed for SDS-PAGE and immunoblotting with indicated antibodies. (**B**) Representative flow cytometry dot plots of 83Mes spheroids treated as in (**A**). Apoptosis was assessed by Annexin V-FITC and propidium iodide (PI) co-staining. Dot plots were generated using FlowJo. (**C**) Quantification of Annexin V positive cells from experiment shown in (**B**). Data represent means ± SEM of 3 independent experiments. Statistical significance was determined by using an ordinary one-way ANOVA followed by Tukey’s multiple comparison test. *, *p* < 0.05; **, *p* < 0.01; ***, *p* < 0.001; ****, *p* < 0.0001.

Our previous studies established that MNK activity sustains translation of oncogenic and anti-apoptotic mRNAs, thereby promoting resilience in mesenchymal GSCs [13]. Further, MNK signaling is induced in response to anticancer therapies, functioning as a pro-survival mechanism that suppresses apoptosis, particularly in the mesenchymal (MES) GBM subtype [25]. To further explore this, we employed the patient-derived GSC line 83Mes, which models the MES GBM subtype [13,39], to assess apoptotic responses following MNK inhibition. As NU1893 blocked neurosphere growth at 5 μM in 83Mes (Figure 6B), we assessed apoptotic signaling at 3 μM. Immunoblot analysis showed that treatment with either SGI-1027 or MNK inhibitor compounds alone did not trigger robust apoptotic signaling (Figure 7A). In contrast, combined treatment with SGI-1027 and either NU808 (Figure 7A, left panels and Figures S4 and S5) or NU1893 (Figure 7A, right panels and Figures S4 and S5) strongly activated apoptotic signaling, as evidenced by greatly increased BAX levels and enhanced cleavage of both Poly(ADP-ribose) polymerase (PARP) and Caspase-3. Flow cytometric analysis of Annexin V staining confirmed these findings (Figure 7B,C). Collectively, these results indicate that our novel and selective MNK inhibitor compounds potently induce antineoplastic effects in GBM with the next-generation compound NU1893 exhibiting superior kinetics over our lead compound NU808. They further support a model in which MNK targeting lends itself well for combinatorial anti-GBM strategies, in particular in highly refractory GSCs of the mesenchymal GBM subtype.

## 4. Discussion

Aberrations in receptor tyrosine kinase (RTK)/MAPK pathways are common across human cancers, and hyperactivation of MAPK signaling contributes to uncontrolled cancer cell growth, survival, migration, and invasion, all of which are considered cancer hallmarks [40]. In GBM, hyperactivated MAPK signaling is frequently observed but pharmacological inhibition of MEK/MAPK pathway components often induces dose-limiting toxicity in preclinical GBM mouse models, limiting therapeutic applicability [41,42]. MNKs represent critical downstream effectors of the MEK/MAPK pathway [43], and their targeting may provide a more tolerable therapeutic strategy because MNK1/2 knockout mice exhibit antitumor effects without obvious adverse effects [14,44]. Consistent with this, mice harboring a non-phosphorylatable eIF4E S209A knock-in mutation show resistance to RAS- and MYC-driven tumorigenesis [45,46], further underscoring the importance of MNK-eIF4E signaling in cancer.

In previous work, we have demonstrated that *MKNK1* expression is elevated in GBM, particularly in the mesenchymal GBM subtype, and that pharmacological targeting of MNKs is a promising strategy that also disrupts GSC self-renewal capacity [13]. Encouraged by these findings, we recently employed medicinal optimization for the development of highly selective MNK inhibitors [18]. This effort led to the identification of NU808 (formerly compound 12 g), which exhibits potent anti-leukemic activity and favorable pharmacokinetic properties, including high brain penetration in mice [18], highlighting its potential utility for central nervous system malignancies. In the present study, we extend these findings by demonstrating potent anti-GBM effects of NU808 and introduce NU1893, a next-generation inhibitor derived from this scaffold.

NU1893 showed a similar kinase selectivity profile as NU808 but displayed enhanced potency in cell-based assays. The greater potency is likely due to enhanced Van der Waals interactions between the bromine and Phe-159 as well as a stronger hydrogen bond interaction between the primary amine and Asp-226, indicating future directions to further improve the potency and selectivity of our MNK inhibitor series. In line with this notion, NU1893 exhibited improved potency over NU808 in terms of inhibition of eIF4E phosphorylation and superior antineoplastic activity across both conventional 2-D viability assays and multipotent 3-D neurosphere models.

We have previously demonstrated that MNK targeting is particularly well suited for combinatorial therapeutic strategies aimed at blocking self-renewal capacity and overcoming resistance in stem-like cancer cell populations [26]. This is largely due to the activation of the MNK-eIF4E axis as a compensatory survival pathway following inhibition of other signaling nodes. For instance, selective inhibition of mechanistic target of rapamycin (mTORC1) by rapalogs induces MNK2 activation in medulloblastoma [27], while arsenic trioxide (ATO) activates MNK1 signaling in GBM [25], both contributing to therapeutic resistance. Thus, pharmacological MNK inhibition represents a rational strategy to enhance the efficacy of combinatorial strategies in brain cancers.

In efforts to identify compounds that synergize with our lead compound NU808 in GBM, we performed compound screens and identified the DNMT inhibitor SGI-1027 as a potent candidate for combination with our novel MNK inhibitor in GBM. SGI-1027 enhanced sensitivity to NU808 across all four cell sources tested, and siRNA-mediated knockdown experiments indicated that these combinatorial antiproliferative effects are primarily mediated through inhibition of MNK1 rather than MNK2. Importantly, similar combinatorial efficacy was observed with the more potent inhibitor NU1893 in 3-D neurosphere models cultured under stem cell-permissive conditions. Further mechanistic studies will be required to fully define the underlying signaling interactions. Still, our observations highlight the relevance of this combinatorial strategy for targeting therapy-resistant GSC subpopulations.

Mesenchymal GSCs exhibit increased therapeutic resistance, and we have previously shown that pharmacological MNK inhibition significantly enhances apoptosis in this GSC subtype, including 83Mes spheroids, when combined with agents such as arsenic trioxide (ATO) [25]. Consistent with this, mesenchymal GSCs displayed pronounced resistance to SGI-1027, requiring approximately tenfold higher concentrations to achieve effects comparable to non-mesenchymal models. This is in line with their established resilience to environmental and therapeutic challenges [13], and underscores the critical role of MNK signaling in sustaining this resistant phenotype [25]. Notably, SGI-1027 alone failed to induce obvious apoptotic responses, whereas cotreatment with NU808 or NU1893 triggered robust apoptosis, with NU1893 again exhibiting superior kinetics. Collectively, these findings demonstrate that MNK inhibition lowers the apoptotic threshold in otherwise refractory GSC populations.

In summary, we report the development of NU1893, a next-generation MNK inhibitor generated through structure guided optimization that demonstrates improved potency and clear advantages over NU808 [18]. Extending beyond our prior work in leukemic models, the current study establishes the efficacy of NU1893 across multiple GBM cell systems, including therapy-resilient glioma stem cell populations. Importantly, we identify pharmacological DNMT inhibition with SGI-1027 as a highly effective combinatorial strategy when combined with MNK targeting. While GBM cell lines and neurospheres derived from the proneural GBM43 PDX line were intrinsically sensitive to SGI-1027, resistance in mesenchymal GSCs can be effectively overcome through combined MNK targeting, resulting in robust induction of apoptosis. Overall, our findings reinforce pharmacological MNK inhibition as a potentially key approach in therapy-resistant mesenchymal GSCs, supporting its integration into combinatorial treatment approaches.

## 5. Conclusions

In conclusion, we report the development and characterization of NU1893, a next-generation selective MNK inhibitor with enhanced potency compared with our previous lead compound NU808. NU1893 effectively suppressed eIF4E phosphorylation, reduced GBM cell viability, impaired neurosphere growth, and inhibited invasive properties in both established and patient-derived GBM models. Our findings further demonstrate that pharmacological inhibition of DNMTs strongly enhances the antineoplastic effects of MNK targeting, particularly in therapy-resilient mesenchymal GSCs, where combined treatment robustly induced apoptosis. Together, these results identify dual MNK and DNMT inhibition as a promising combinatorial therapeutic strategy for GBM and further support MNK signaling as clinically relevant vulnerability in aggressive and treatment-resistant GSC populations.

## Data Availability

The data that support the findings of this study are available in the main text or the Supplemental Materials. Values for all data points in graphs are reported in Table S4.

## Supplementary Materials

The following supporting information can be downloaded at: https://www.mdpi.com/article/10.3390/cancers18132086/s1, Table S1: Key resources; Table S2: KINOMEscan^TM^; Table S3: Compound screen; Table S4: Supporting data values. Supplementary information: Uncropped immunoblots and NMR spectra of NU1893. Figure S1A. Uncropped immunoblots related to Figure 2A,B. Figure S1B. Uncropped immunoblots related to Figure 2A,B. Figure S2A. Uncropped immunoblots related to Figure 3A,B. Figure S2B. Uncropped immunoblots related to Figure 3A,B. Figure S3. Uncropped immunoblots related to Figure 5I. Figure S4A,B. Uncropped immunoblots related to Figure 7A left panels. Figure S4C. Uncropped immunoblots related to Figure 7A left panels. Figure S4D. Uncropped immunoblots related to Figure 7A left panels. Figure S4E. Uncropped immunoblots related to Figure 7A left panels. Figure S4F. Uncropped immunoblots related to Figure 7A left panels. Figure S5A. Uncropped immunoblots related to Figure 7A right panels. Figure S5B. Uncropped immunoblots related to Figure 7A right panels. Figure S5C. Uncropped immunoblots related to Figure 7A right panels. Figure S5D. Uncropped immunoblots related to Figure 7A right panels. Figure S5E. Uncropped immunoblots related to Figure 7A right panels. Figure S5F. Uncropped immunoblots related to Figure 7A right panels. Figure S6. NMR spectra for NU1893. ^1^H (top) and ^13^C (bottom) NMR spectra in CD_3_OD. In the ^13^C NMR spectrum, a zoomed in section for aromatic peaks is shown for clarity.

## Abbreviations

The following abbreviations are used in this manuscript:

GBM	Glioblastoma
GSCs	Glial stem cells
BBB	Blood–brain barrier
MAPK	Mitogen-activated protein kinase
MNK	Mitogen-activated protein kinase interacting kinases
eIF4E	Eukaryotic initiation factor 4E
hnRNPA1	Heterogeneous nuclear ribonucleoprotein A1
cPLA2	Cytosolic phospholipase A2
PSF	Pre-mRNA splicing factor
DNMTs	DNA methyltransferases
PDX	Patient-derived xenograft
CSC	Cancer stem cell
VC	Vehicle control
PDB	Protein Data Bank
MES	Mesenchymal
PARP	Poly(ADP-ribose) polymerase
RTK	Receptor tyrosine kinase
mTORC1	Mechanistic target of rapamycin C1
ATO	Arsenic trioxide

## References

[1] Stupp R., Mason W.P., van den Bent M.J., Weller M., Fisher B., Taphoorn M.J., Belanger K., Brandes A.A., Marosi C., Bogdahn U. (2005). Radiotherapy plus concomitant and adjuvant temozolomide for glioblastoma. N. Engl. J. Med..

[2] Price M., Ballard C.A.P., Benedetti J.R., Kruchko C., Barnholtz-Sloan J.S., Ostrom Q.T. (2025). CBTRUS Statistical Report: Primary Brain and Other Central Nervous System Tumors Diagnosed in the United States in 2018–2022. Neuro Oncol..

[3] Kisby T., Borst G.R., Coope D.J., Kostarelos K. (2025). Targeting the glioblastoma resection margin with locoregional nanotechnologies. Nat. Rev. Clin. Oncol..

[4] Xu C., Hou P., Li X., Xiao M., Zhang Z., Li Z., Xu J., Liu G., Tan Y., Fang C. (2024). Comprehensive understanding of glioblastoma molecular phenotypes: Classification, characteristics, and transition. Cancer Biol. Med..

[5] Yabo Y.A., Niclou S.P., Golebiewska A. (2022). Cancer cell heterogeneity and plasticity: A paradigm shift in glioblastoma. Neuro Oncol..

[6] Khasraw M., Fujita Y., Lee-Chang C., Balyasnikova I.V., Najem H., Heimberger A.B. (2021). New Approaches to Glioblastoma. Annu. Rev. Med..

[7] Mitchell K., Troike K., Silver D.J., Lathia J.D. (2020). The evolution of the cancer stem cell state in glioblastoma—Emerging insights into the next-generation of functional interactions. Neuro Oncol..

[8] Gimple R.C., Yang K., Halbert M.E., Agnihotri S., Rich J.N. (2022). Brain cancer stem cells: Resilience through adaptive plasticity and hierarchical heterogeneity. Nat. Rev. Cancer.

[9] Gimple R.C., Bhargava S., Dixit D., Rich J.N. (2019). Glioblastoma stem cells: Lessons from the tumor hierarchy in a lethal cancer. Genes Dev..

[10] Lathia J.D., Mack S.C., Mulkearns-Hubert E.E., Valentim C.L., Rich J.N. (2015). Cancer stem cells in glioblastoma. Genes Dev..

[11] Chouleur T., Tremblay M.L., Bikfalvi A. (2020). Mechanisms of invasion in glioblastoma. Curr. Opin. Oncol..

[12] Aitchison E.E., Dimesa A.M., Shoari A. (2025). Matrix Metalloproteinases in Glioma: Drivers of Invasion and Therapeutic Targets. BioTech.

[13] Bell J.B., Eckerdt F.D., Alley K., Magnusson L.P., Hussain H., Bi Y., Arslan A.D., Clymer J., Alvarez A.A., Goldman S. (2016). MNK Inhibition Disrupts Mesenchymal Glioma Stem Cells and Prolongs Survival in a Mouse Model of Glioblastoma. Mol. Cancer Res..

[14] Ueda T., Watanabe-Fukunaga R., Fukuyama H., Nagata S., Fukunaga R. (2004). Mnk2 and Mnk1 are essential for constitutive and inducible phosphorylation of eukaryotic initiation factor 4E but not for cell growth or development. Mol. Cell Biol..

[15] Xu W., Kannan S., Verma C.S., Nacro K. (2022). Update on the Development of MNK Inhibitors as Therapeutic Agents. J. Med. Chem..

[16] Guo Q., Li V.Z., Nichol J.N., Huang F., Yang W., Preston S.E.J., Talat Z., Lefrere H., Yu H., Zhang G. (2019). MNK1/NODAL Signaling Promotes Invasive Progression of Breast Ductal Carcinoma In Situ. Cancer Res..

[17] Kwegyir-Afful A.K., Bruno R.D., Purushottamachar P., Murigi F.N., Njar V.C. (2016). Galeterone and VNPT55 disrupt Mnk-eIF4E to inhibit prostate cancer cell migration and invasion. FEBS J..

[18] Vagadia P.P., Izquierdo-Ferrer J., Mazewski C., Blyth G., Beauchamp E.M., Clutter M.R., Stern C.L., Mishra R.K., Nahotko D., Small S. (2025). Discovery of Potent and Selective MNK Kinase Inhibitors for the Treatment of Leukemia. J. Med. Chem..

[19] Mishra R.K., Clutter M.R., Blyth G.T., Kosciuczuk E.M., Blackburn A.Z., Beauchamp E.M., Schiltz G.E., Platanias L.C. (2019). Discovery of novel Mnk inhibitors using mutation-based induced-fit virtual high-throughput screening. Chem. Biol. Drug Des..

[20] Fernandez A., Monsen P.J., Platanias L.C., Schiltz G.E. (2023). Medicinal chemistry approaches to target the MNK-eIF4E axis in cancer. RSC Med. Chem..

[21] Yang X., Liu Z., Yin X., Zeng Y., Guo G. (2022). Inhibition MNK-eIF4E-beta-catenin preferentially sensitizes gastric cancer to chemotherapy. Fundam. Clin. Pharmacol..

[22] Liu S., Zha J., Lei M. (2018). Inhibiting ERK/Mnk/eIF4E broadly sensitizes ovarian cancer response to chemotherapy. Clin. Transl. Oncol..

[23] Grzmil M., Seebacher J., Hess D., Behe M., Schibli R., Moncayo G., Frank S., Hemmings B.A. (2016). Inhibition of MNK pathways enhances cancer cell response to chemotherapy with temozolomide and targeted radionuclide therapy. Cell. Signal..

[24] Teo T., Yu M., Yang Y., Gillam T., Lam F., Sykes M.J., Wang S. (2015). Pharmacologic co-inhibition of Mnks and mTORC1 synergistically suppresses proliferation and perturbs cell cycle progression in blast crisis-chronic myeloid leukemia cells. Cancer Lett..

[25] Bell J.B., Eckerdt F., Dhruv H.D., Finlay D., Peng S., Kim S., Kroczynska B., Beauchamp E.M., Alley K., Clymer J. (2018). Differential Response of Glioma Stem Cells to Arsenic Trioxide Therapy Is Regulated by MNK1 and mRNA Translation. Mol. Cancer Res..

[26] Eckerdt F., Bell J.B., Beauchamp E.M., Clymer J., Blyth G.T., Kosciuczuk E.M., Ma Q., Chen D.Z., Horbinski C., Goldman S. (2019). Potent Antineoplastic Effects of Combined PI3Kalpha-MNK Inhibition in Medulloblastoma. Mol. Cancer Res..

[27] Stead R.L., Proud C.G. (2013). Rapamycin enhances eIF4E phosphorylation by activating MAP kinase-interacting kinase 2a (Mnk2a). FEBS Lett..

[28] Wang K., He Z., Jin G., Jin S., Du Y., Yuan S., Zhang J. (2024). Targeting DNA methyltransferases for cancer therapy. Bioorg. Chem..

[29] Armoundas A.A., Piperi C. (2026). DNMT1 drives glioma progression and modulates therapy response: A novel therapeutic target. Eur. J. Med. Chem..

[30] Eckerdt F.D., Bell J.B., Gonzalez C., Oh M.S., Perez R.E., Mazewski C., Fischietti M., Goldman S., Nakano I., Platanias L.C. (2020). Combined PI3Kalpha-mTOR Targeting of Glioma Stem Cells. Sci. Rep..

[31] Eckerdt F., Alvarez A., Bell J., Arvanitis C., Iqbal A., Arslan A.D., Hu B., Cheng S.Y., Goldman S., Platanias L.C. (2016). A simple, low-cost staining method for rapid-throughput analysis of tumor spheroids. Biotechniques.

[32] Arslan A.D., Sassano A., Saleiro D., Lisowski P., Kosciuczuk E.M., Fischietti M., Eckerdt F., Fish E.N., Platanias L.C. (2017). Human SLFN5 is a transcriptional co-repressor of STAT1-mediated interferon responses and promotes the malignant phenotype in glioblastoma. Oncogene.

[33] Vinci M., Gowan S., Boxall F., Patterson L., Zimmermann M., Court W., Lomas C., Mendiola M., Hardisson D., Eccles S.A. (2012). Advances in establishment and analysis of three-dimensional tumor spheroid-based functional assays for target validation and drug evaluation. BMC Biol..

[34] Xu X., Farach-Carson M.C., Jia X. (2014). Three-dimensional in vitro tumor models for cancer research and drug evaluation. Biotechnol. Adv..

[35] Campos B., Gal Z., Baader A., Schneider T., Sliwinski C., Gassel K., Bageritz J., Grabe N., von Deimling A., Beckhove P. (2014). Aberrant self-renewal and quiescence contribute to the aggressiveness of glioblastoma. J. Pathol..

[36] Laks D.R., Crisman T.J., Shih M.Y., Mottahedeh J., Gao F., Sperry J., Garrett M.C., Yong W.H., Cloughesy T.F., Liau L.M. (2016). Large-scale assessment of the gliomasphere model system. Neuro Oncol..

[37] Mair D.B., Ames H.M., Li R. (2018). Mechanisms of invasion and motility of high-grade gliomas in the brain. Mol. Biol. Cell.

[38] Juarez-Mercado K.E., Prieto-Martinez F.D., Sanchez-Cruz N., Pena-Castillo A., Prada-Gracia D., Medina-Franco J.L. (2020). Expanding the Structural Diversity of DNA Methyltransferase Inhibitors. Pharmaceuticals.

[39] Mao P., Joshi K., Li J., Kim S.H., Li P., Santana-Santos L., Luthra S., Chandran U.R., Benos P.V., Smith L. (2013). Mesenchymal glioma stem cells are maintained by activated glycolytic metabolism involving aldehyde dehydrogenase 1A3. Proc. Natl. Acad. Sci. USA.

[40] Hanahan D., Weinberg R.A. (2011). Hallmarks of cancer: The next generation. Cell.

[41] Prados M.D., Byron S.A., Tran N.L., Phillips J.J., Molinaro A.M., Ligon K.L., Wen P.Y., Kuhn J.G., Mellinghoff I.K., de Groot J.F. (2015). Toward precision medicine in glioblastoma: The promise and the challenges. Neuro Oncol..

[42] McNeill R.S., Canoutas D.A., Stuhlmiller T.J., Dhruv H.D., Irvin D.M., Bash R.E., Angus S.P., Herring L.E., Simon J.M., Skinner K.R. (2017). Combination therapy with potent PI3K and MAPK inhibitors overcomes adaptive kinome resistance to single agents in preclinical models of glioblastoma. Neuro Oncol..

[43] Diab S., Kumarasiri M., Yu M., Teo T., Proud C., Milne R., Wang S. (2014). MAP Kinase-Interacting Kinases-Emerging Targets against Cancer. Chem. Biol..

[44] Ueda T., Sasaki M., Elia A.J., Chio I.I.C., Hamada K., Fukunaga R., Mak T.W. (2010). Combined deficiency for MAP kinase-interacting kinase 1 and 2 (Mnk1 and Mnk2) delays tumor development. Proc. Natl. Acad. Sci. USA.

[45] Wendel H.G., Silva R.L., Malina A., Mills J.R., Zhu H., Ueda T., Watanabe-Fukunaga R., Fukunaga R., Teruya-Feldstein J., Pelletier J. (2007). Dissecting eIF4E action in tumorigenesis. Genes Dev..

[46] Furic L., Rong L., Larsson O., Koumakpayi I.H., Yoshida K., Brueschke A., Petroulakis E., Robichaud N., Pollak M., Gaboury L.A. (2010). eIF4E phosphorylation promotes tumorigenesis and is associated with prostate cancer progression. Proc. Natl. Acad. Sci. USA.

