# Supplementary material for: Potent Anti-Glioblastoma Effects of Next-Generation MNK Inhibitors
Source: Cancers (Basel). 2026 Jun 27;18(13):2086. doi: 10.3390/cancers18132086 (PMC13360561; doi:10.3390/cancers18132086)
Supplement: Supplementary file 1 [file cancers-18-02086-s001.zip › Supplementary Figures revision.pdf]

A

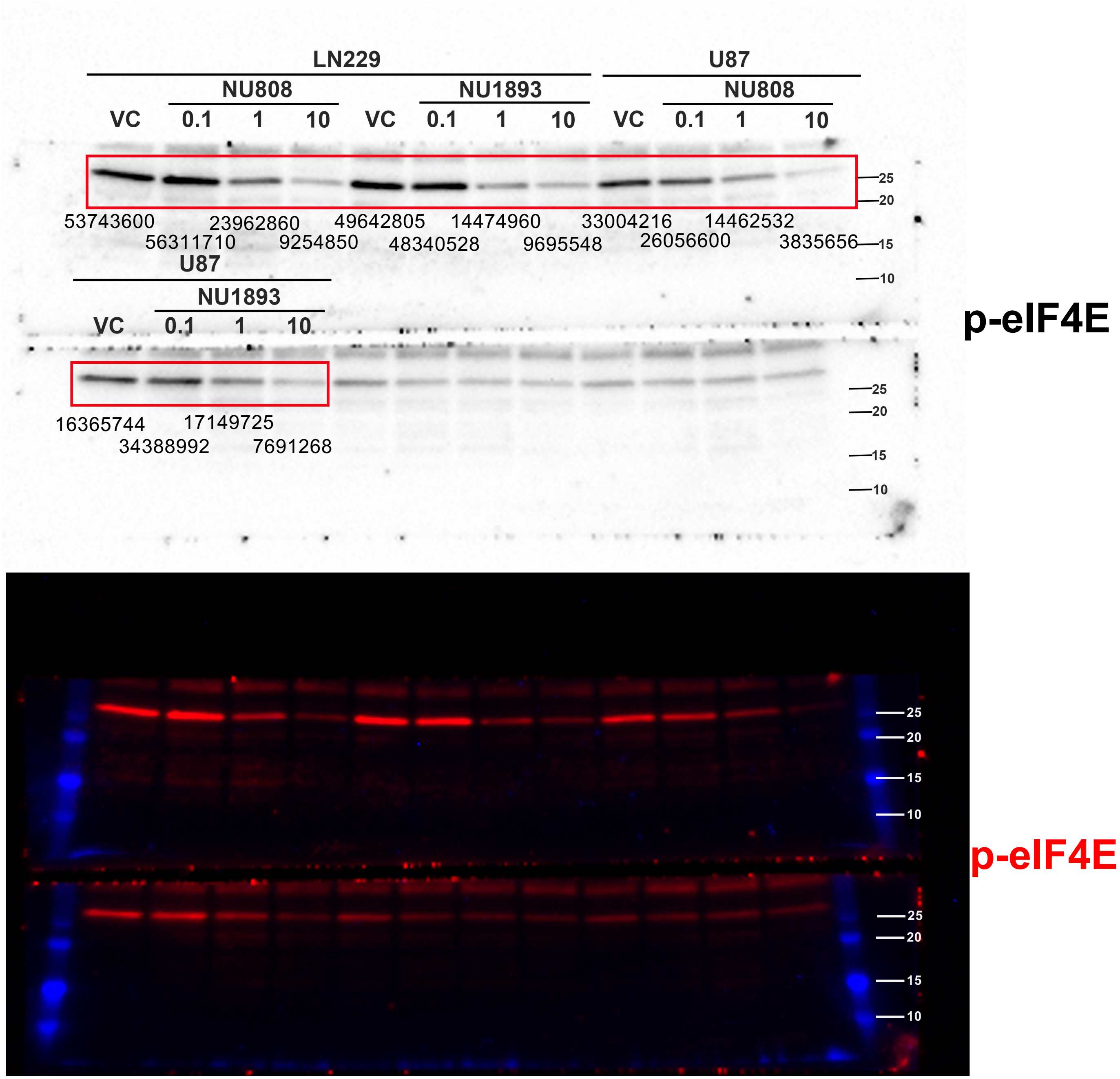

Figure S1A. Uncropped immunoblots related to **Figure 2A,B.** Immunoblot for p-eIF4E (S209) of single chemiluminescence channel (upper) and multichannel with MW marker (lower). Immunoblot images were acquired using a ChemiDoc imaging system (Bio-Rad), and densitometric analysis was performed with Image Lab software (Bio-Rad). Band intensities were quantified using the "Adjusted Volume (Int)" values generated by the software, which are indicated below the respective bands.

B

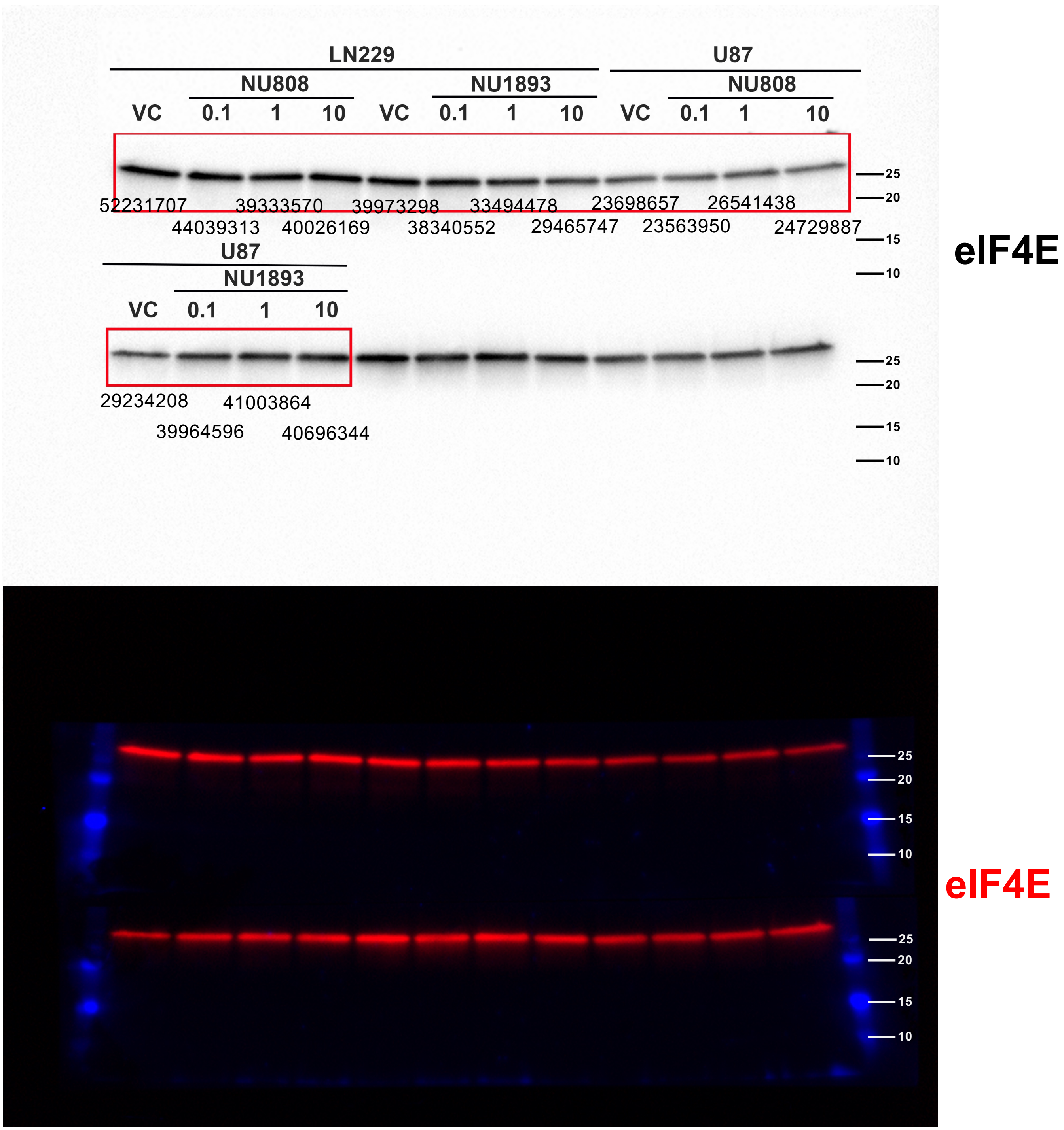

Figure S1B. Uncropped immunoblots related to **Figure 2A,B.** Immunoblot for eIF4E of single chemiluminescence channel (upper) and multichannel with MW marker (lower). Immunoblot images were acquired using a ChemiDoc imaging system (Bio-Rad), and densitometric analysis was performed with Image Lab software (Bio-Rad). Band intensities were quantified using the "Adjusted Volume (Int)" values generated by the software, which are indicated below the respective bands.

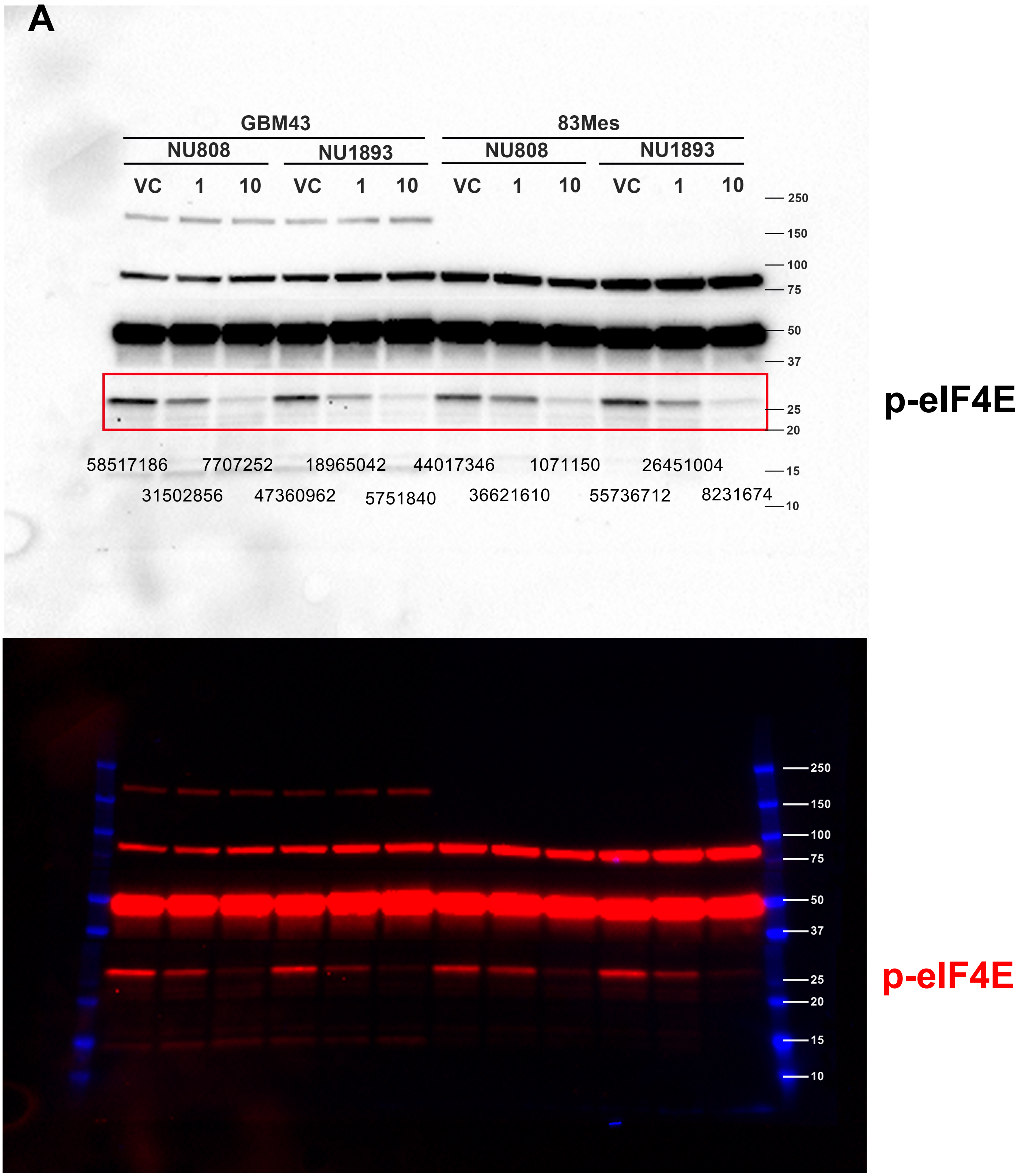

Figure S2A. Uncropped immunoblots related to **Figure 3A,B.** Immunoblot for p-eIF4E (S209) of single chemiluminescence channel (upper) and multichannel with MW marker (lower). Immunoblot images were acquired using a ChemiDoc imaging system (Bio-Rad), and densitometric analysis was performed with Image Lab software (Bio-Rad). Band intensities were quantified using the "Adjusted Volume (Int)" values generated by the software, which are indicated below the respective bands.

B

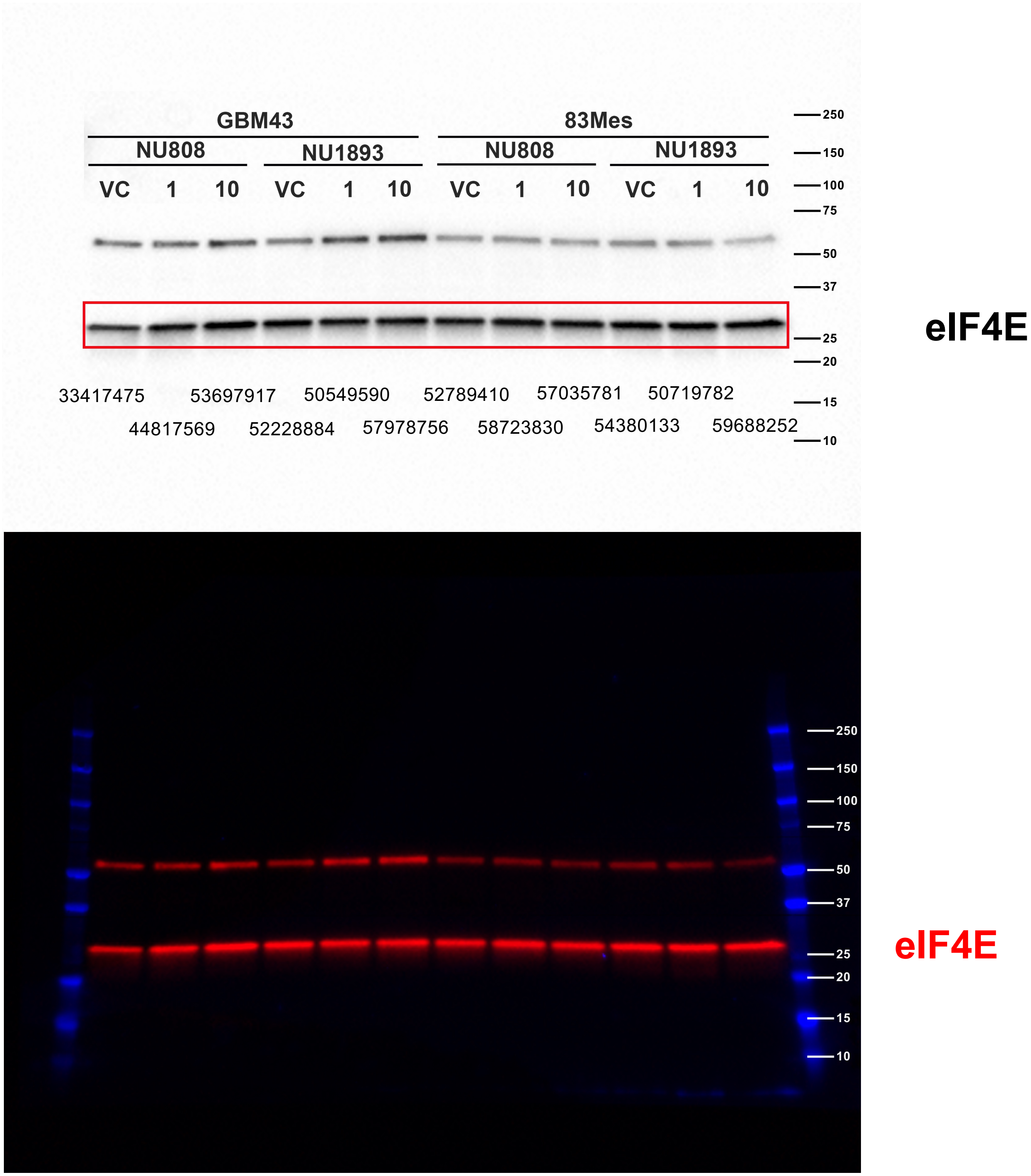

Figure S2B. Uncropped immunoblots related to **Figure 3A,B.** Immunoblot for eIF4E of single chemiluminescence channel (upper) and multichannel with MW marker (lower). Immunoblot images were acquired using a ChemiDoc imaging system (Bio-Rad), and densitometric analysis was performed with Image Lab software (Bio-Rad). Band intensities were quantified using the "Adjusted Volume (Int)" values generated by the software, which are indicated below the respective bands.

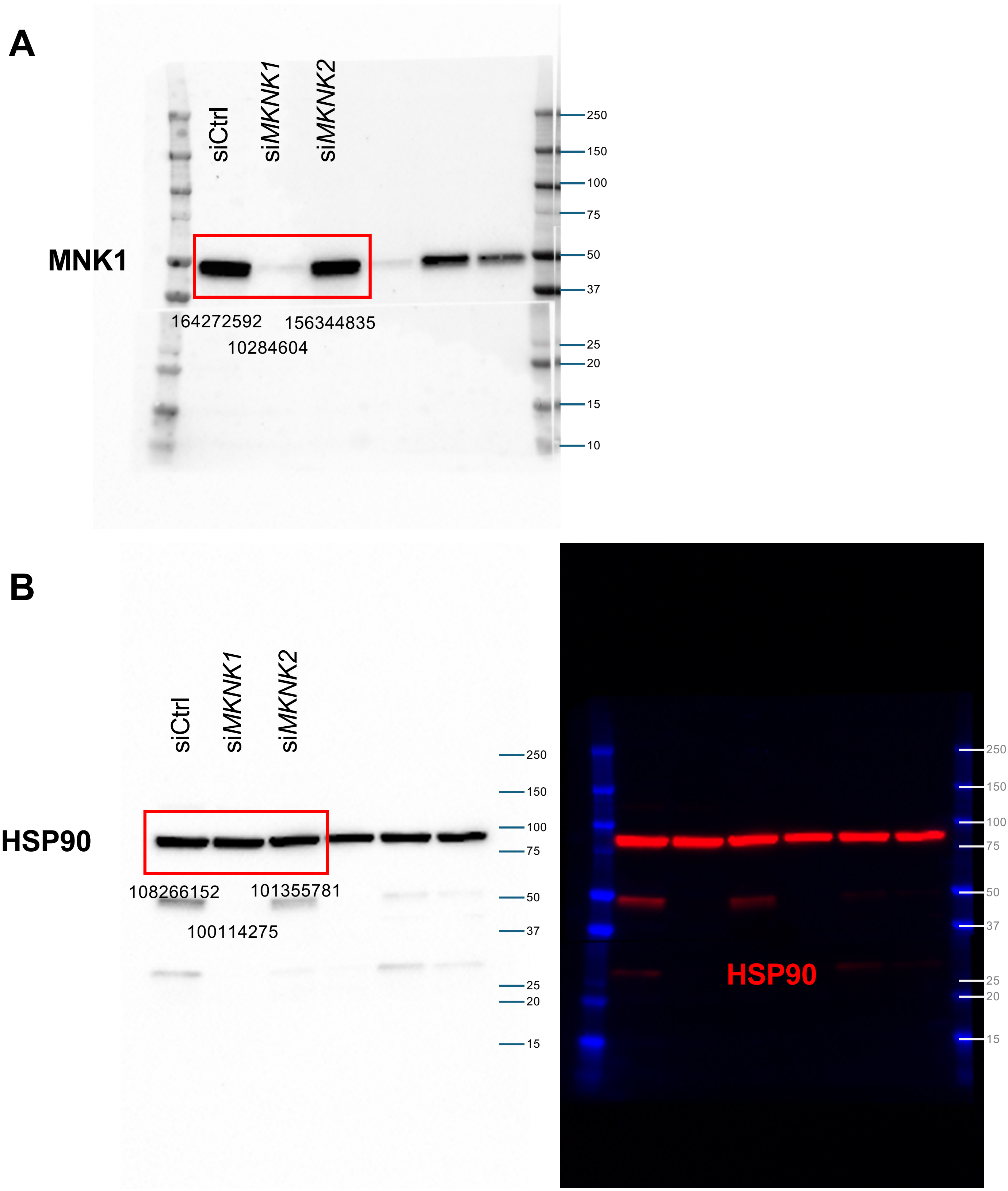

Figure S3. Uncropped immunoblots related to **Figure 5I.** Immunoblot for MNK1 **(A)** and HSP90 **(B)** of single chemiluminescence channel (left) and multichannel with MW marker (right). Immunoblot images were acquired using a ChemiDoc imaging system (Bio-Rad), and densitometric analysis was performed with Image Lab software (Bio-Rad). Band intensities were quantified using the "Adjusted Volume (Int)" values generated by the software, which are indicated below the respective bands.

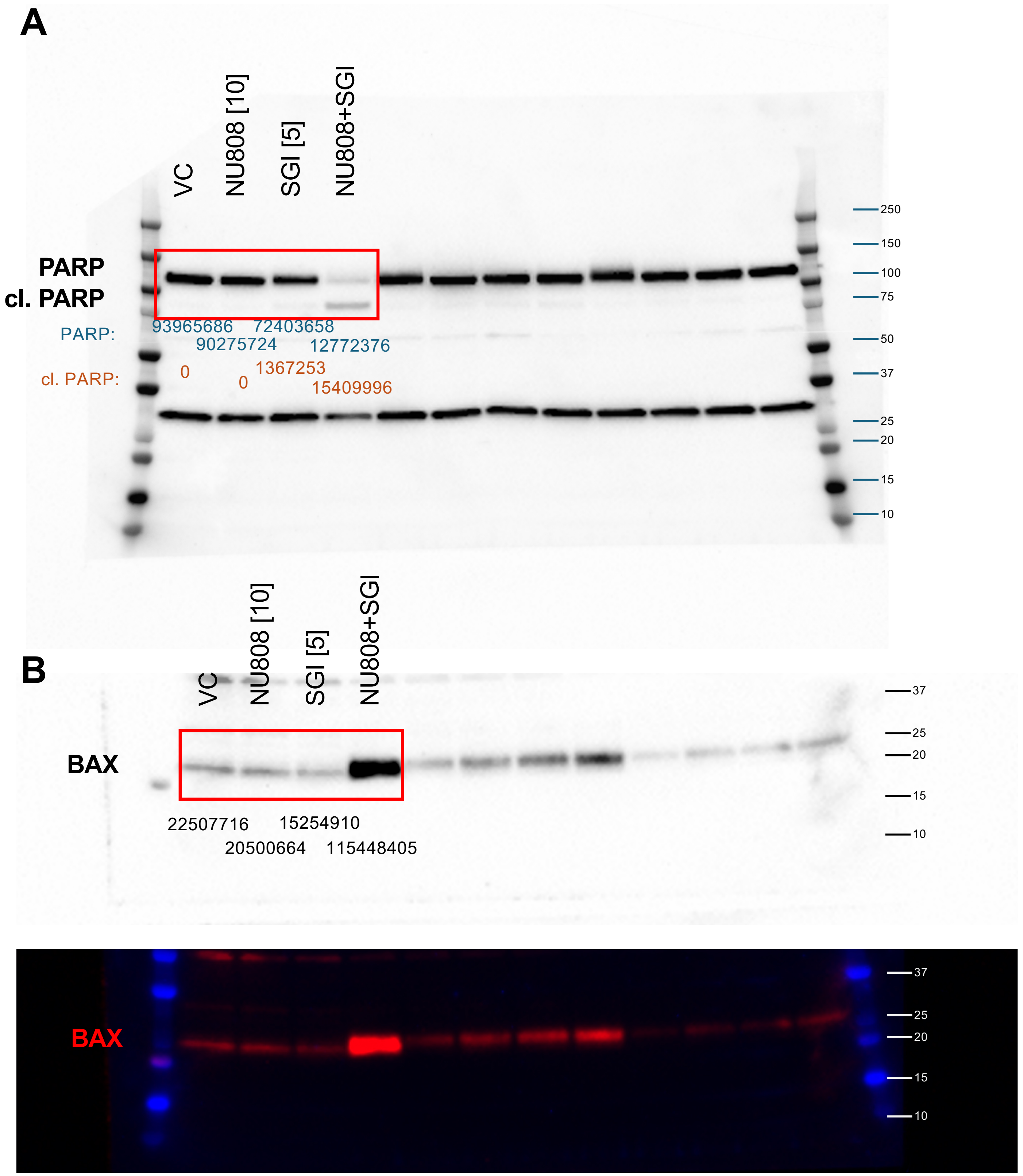

Figure S4A,B. Uncropped immunoblots related to **Figure 7A left panels.** Immunoblot for PARP **(A)** and BAX **(B)** of single chemiluminescence channel (upper) and multichannel with MW marker (lower). Immunoblot images were acquired using a ChemiDoc imaging system (Bio-Rad), and densitometric analysis was performed with Image Lab software (Bio-Rad). Band intensities were quantified using the "Adjusted Volume (Int)" values generated by the software, which are indicated below the respective bands.

C

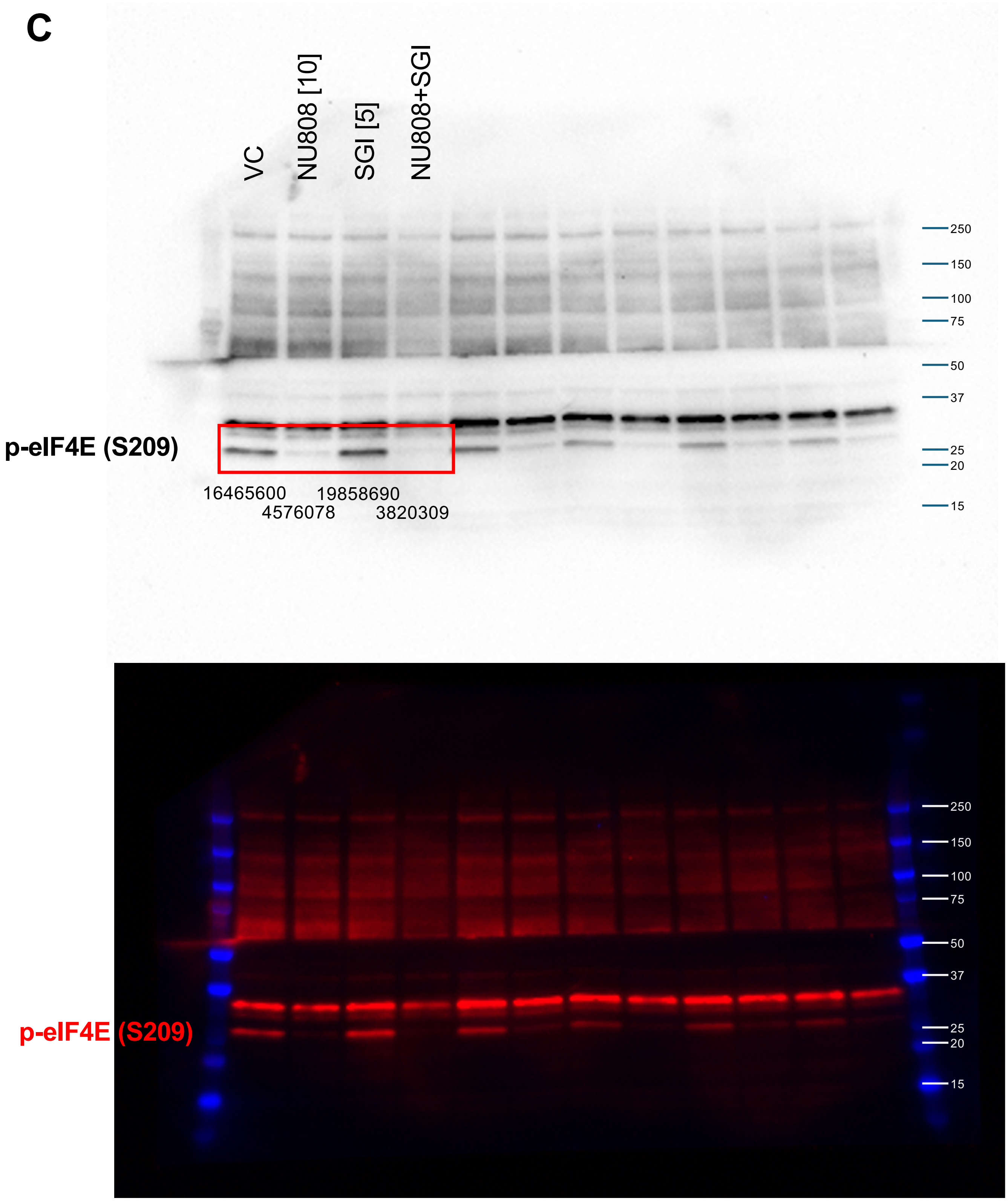

Figure S4C. Uncropped immunoblots related to **Figure 7A left panels.** Immunoblot for p-eIF4E (S209) of single chemiluminescence channel (upper) and multichannel with MW marker (lower). Immunoblot images were acquired using a ChemiDoc imaging system (Bio-Rad), and densitometric analysis was performed with Image Lab software (Bio-Rad). Band intensities were quantified using the "Adjusted Volume (Int)" values generated by the software, which are indicated below the respective bands.

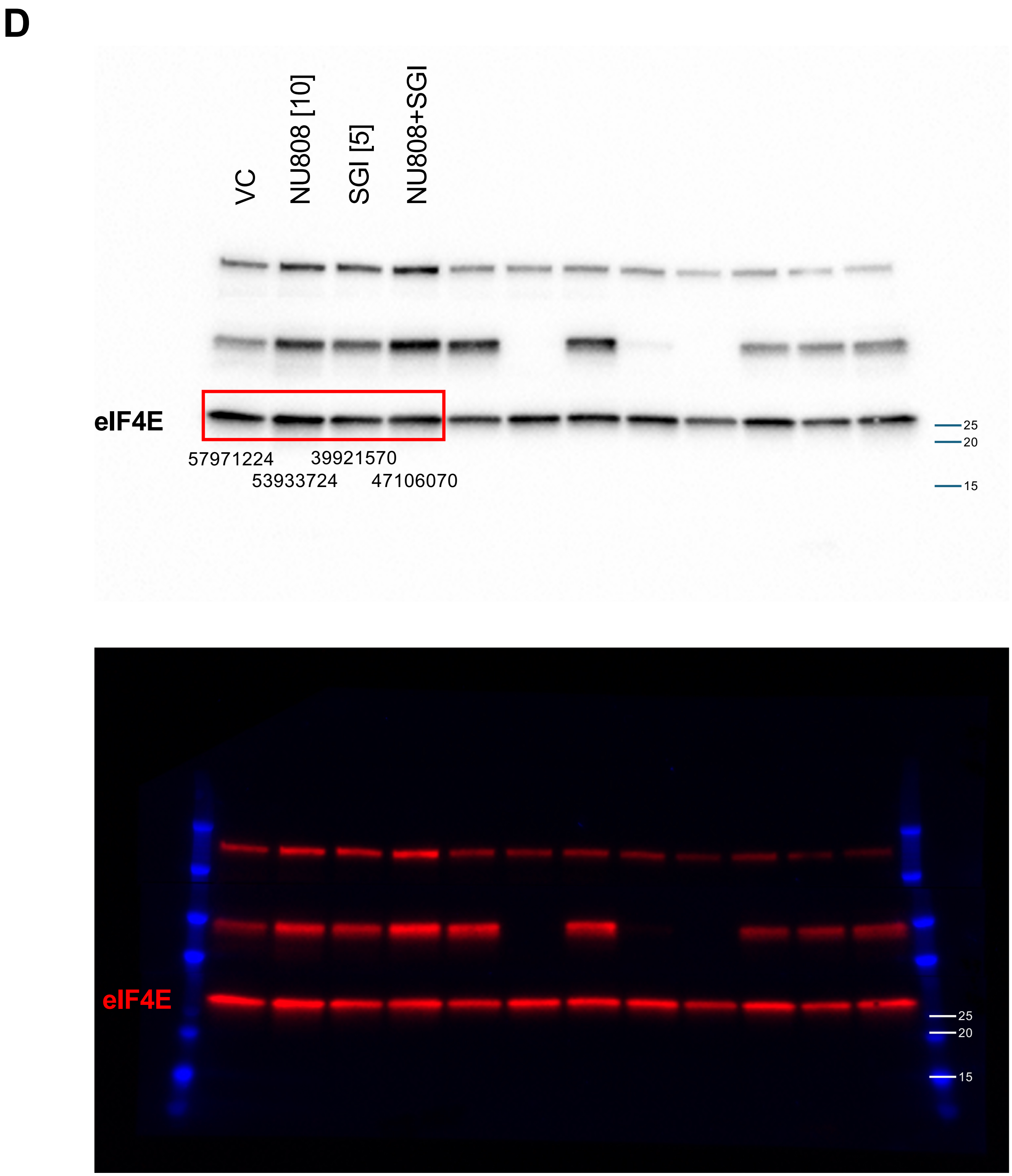

Figure S4D. Uncropped immunoblots related to **Figure 7A left panels.** Immunoblot for eIF4E of single chemiluminescence channel (upper) and multichannel with MW marker (lower). Immunoblot images were acquired using a ChemiDoc imaging system (Bio-Rad), and densitometric analysis was performed with Image Lab software (Bio-Rad). Band intensities were quantified using the “Adjusted Volume (Int)” values generated by the software, which are indicated below the respective bands.

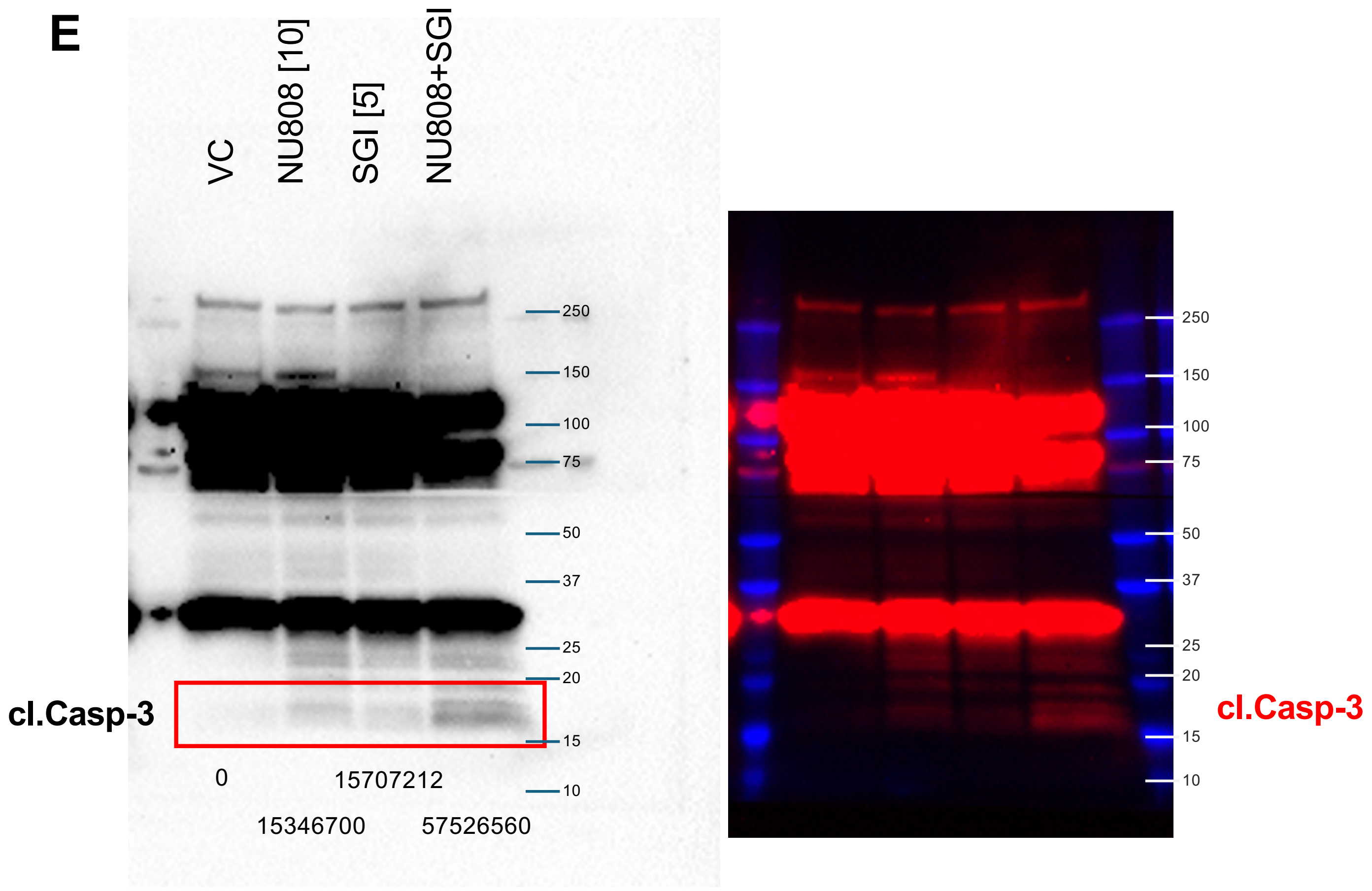

Figure S4E. Uncropped immunoblots related to **Figure 7A left panels.** Immunoblot for cleaved Caspase 3 of single chemiluminescence channel (left) and multichannel with MW marker (right). Immunoblot images were acquired using a ChemiDoc imaging system (Bio-Rad), and densitometric analysis was performed with Image Lab software (Bio-Rad). Band intensities were quantified using the "Adjusted Volume (Int)" values generated by the software, which are indicated below the respective bands.

F

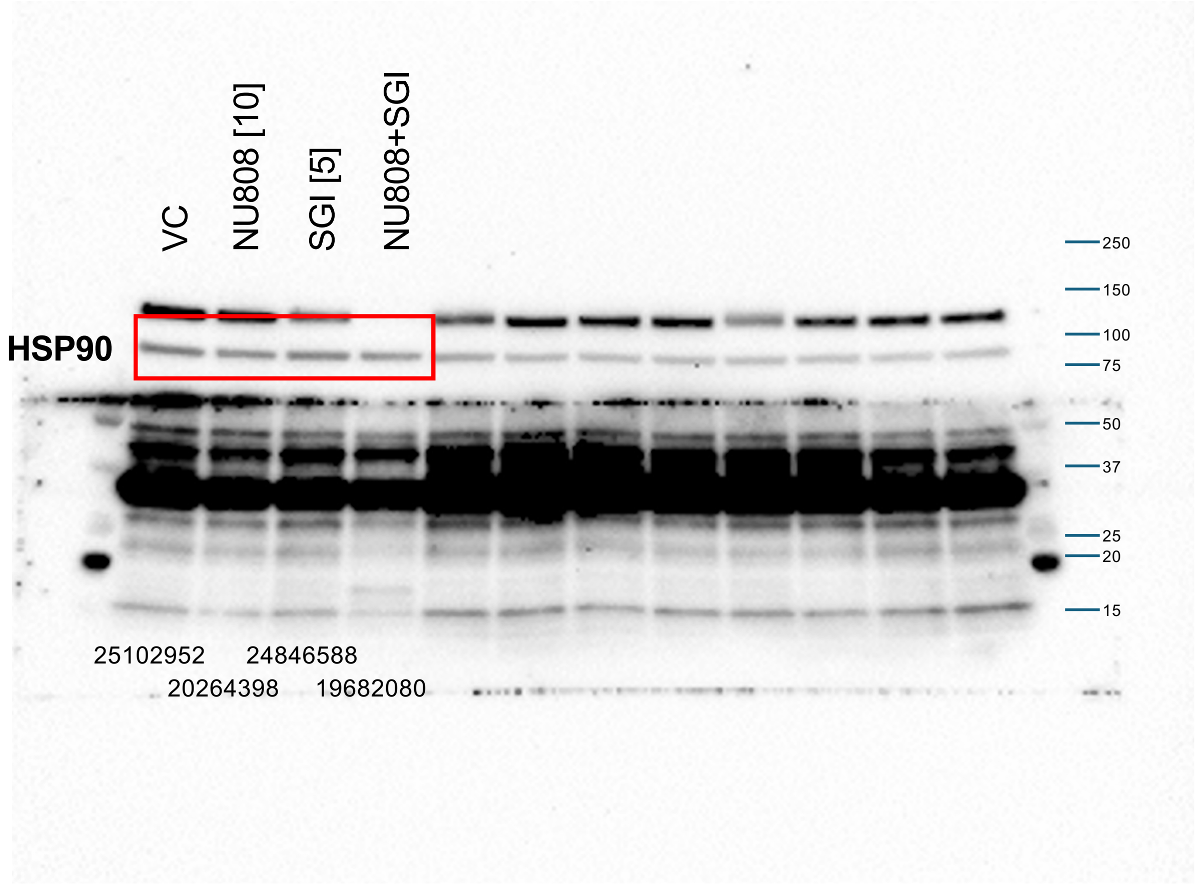

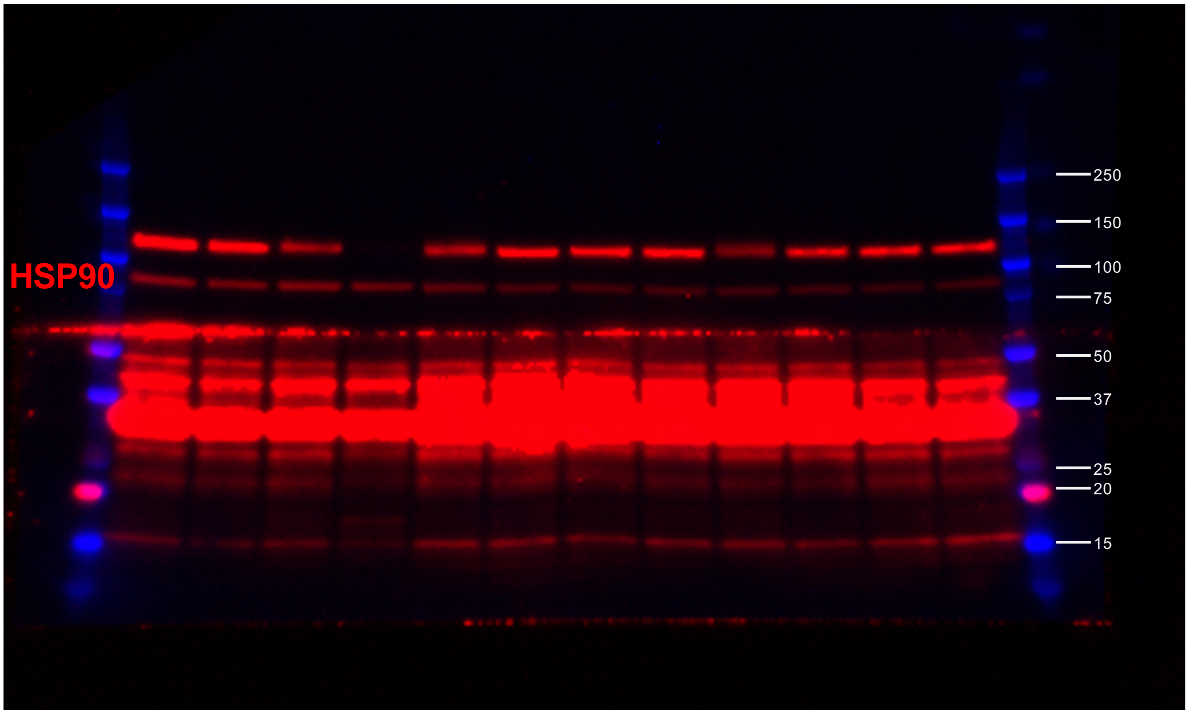

Figure S4F. Uncropped immunoblots related to **Figure 7A left panels.** Immunoblot for HSP90 of single chemiluminescence channel (upper) and multichannel with MW marker (lower). Immunoblot images were acquired using a ChemiDoc imaging system (Bio-Rad), and densitometric analysis was performed with Image Lab software (Bio-Rad). Band intensities were quantified using the "Adjusted Volume (Int)" values generated by the software, which are indicated below the respective bands.

A

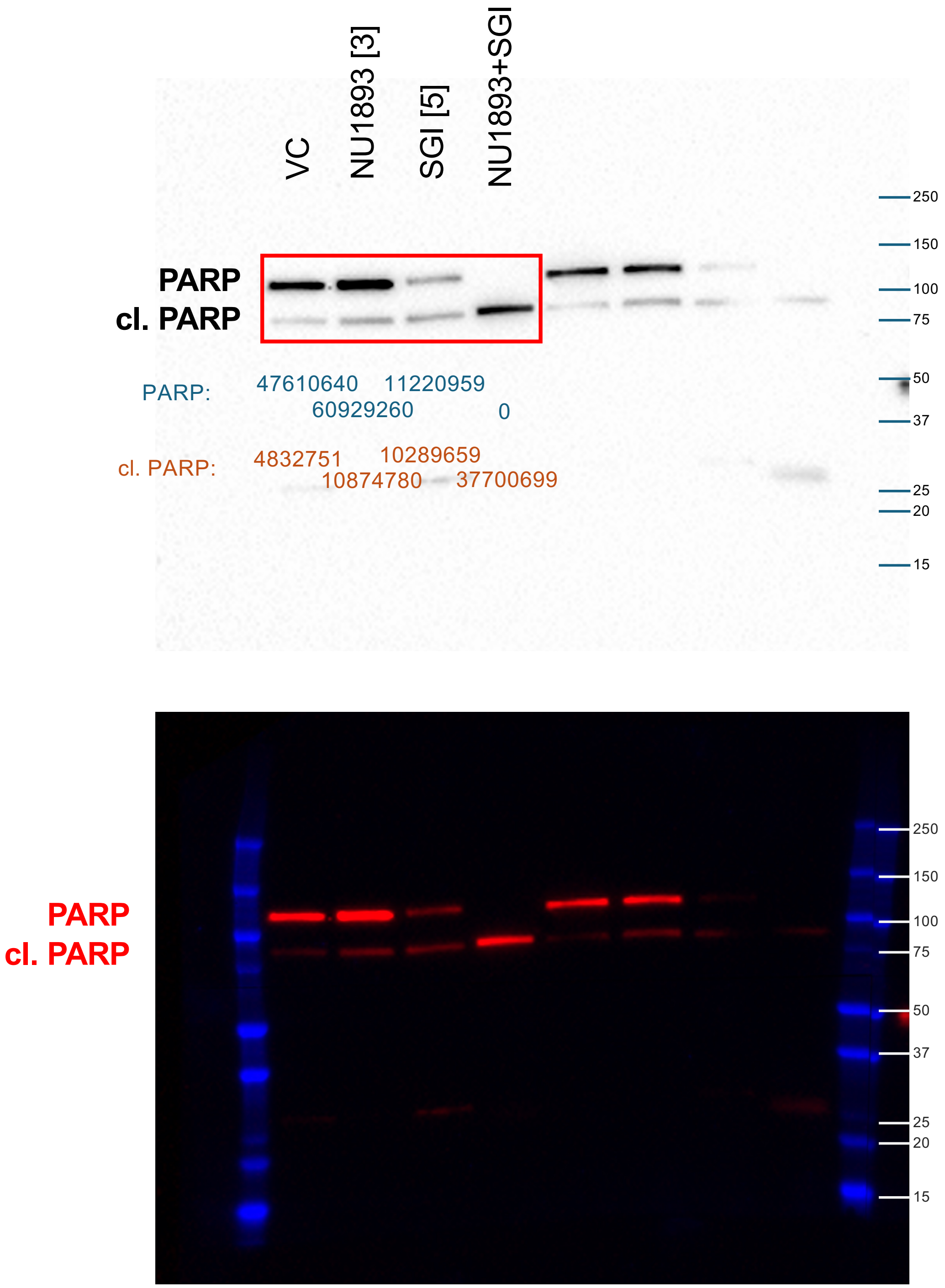

Figure S5A. Uncropped immunoblots related to **Figure 7A right panels.** Immunoblot for PARP of single chemiluminescence channel (upper) and multichannel with MW marker (lower). Immunoblot images were acquired using a ChemiDoc imaging system (Bio-Rad), and densitometric analysis was performed with Image Lab software (Bio-Rad). Band intensities were quantified using the “Adjusted Volume (Int)” values generated by the software, which are indicated below the respective bands.

B

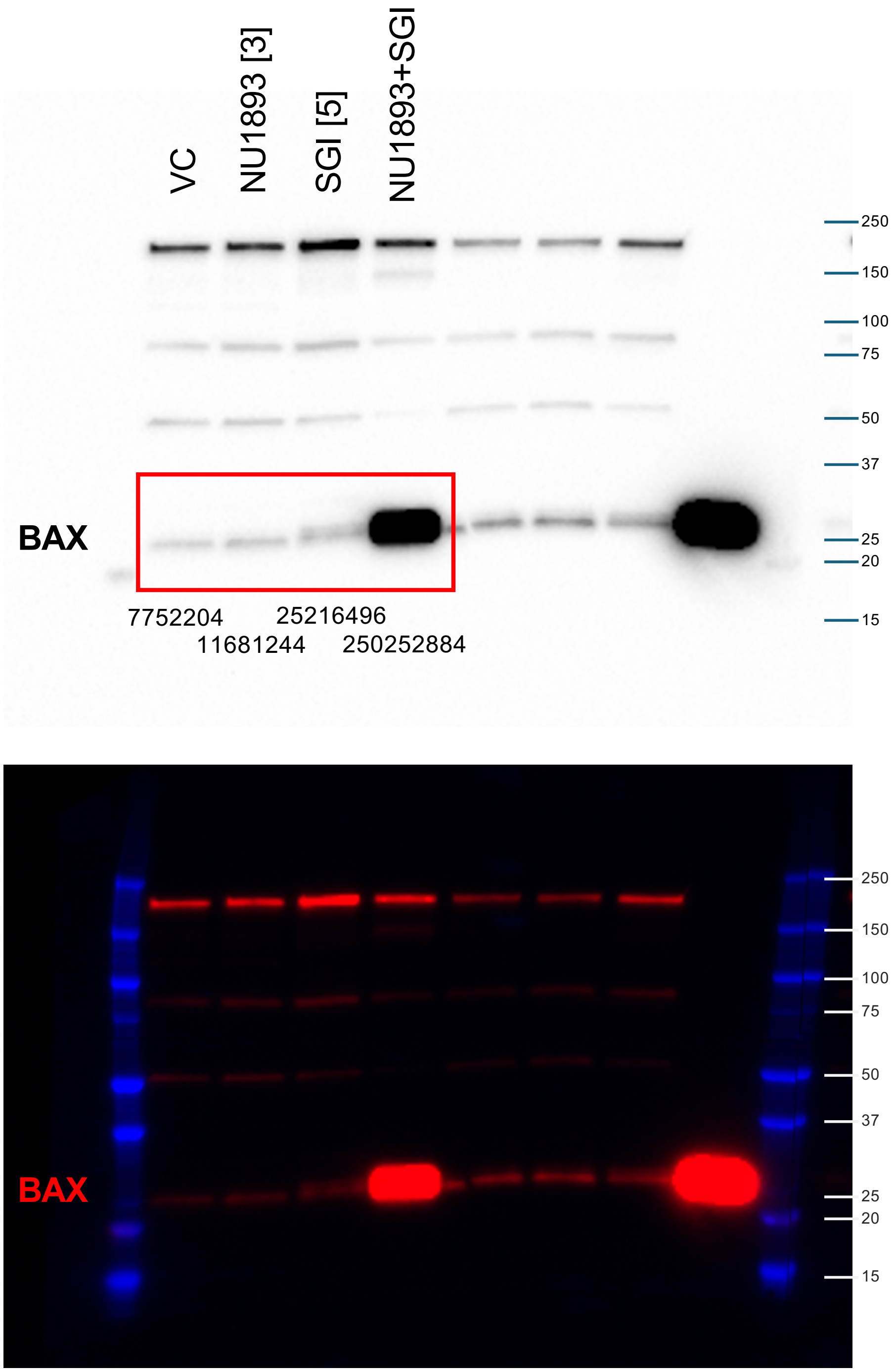

Figure S5B. Uncropped immunoblots related to **Figure 7A right panels.** Immunoblot for BAX of single chemiluminescence channel (upper) and multichannel with MW marker (lower). Immunoblot images were acquired using a ChemiDoc imaging system (Bio-Rad), and densitometric analysis was performed with Image Lab software (Bio-Rad). Band intensities were quantified using the "Adjusted Volume (Int)" values generated by the software, which are indicated below the respective bands.

C

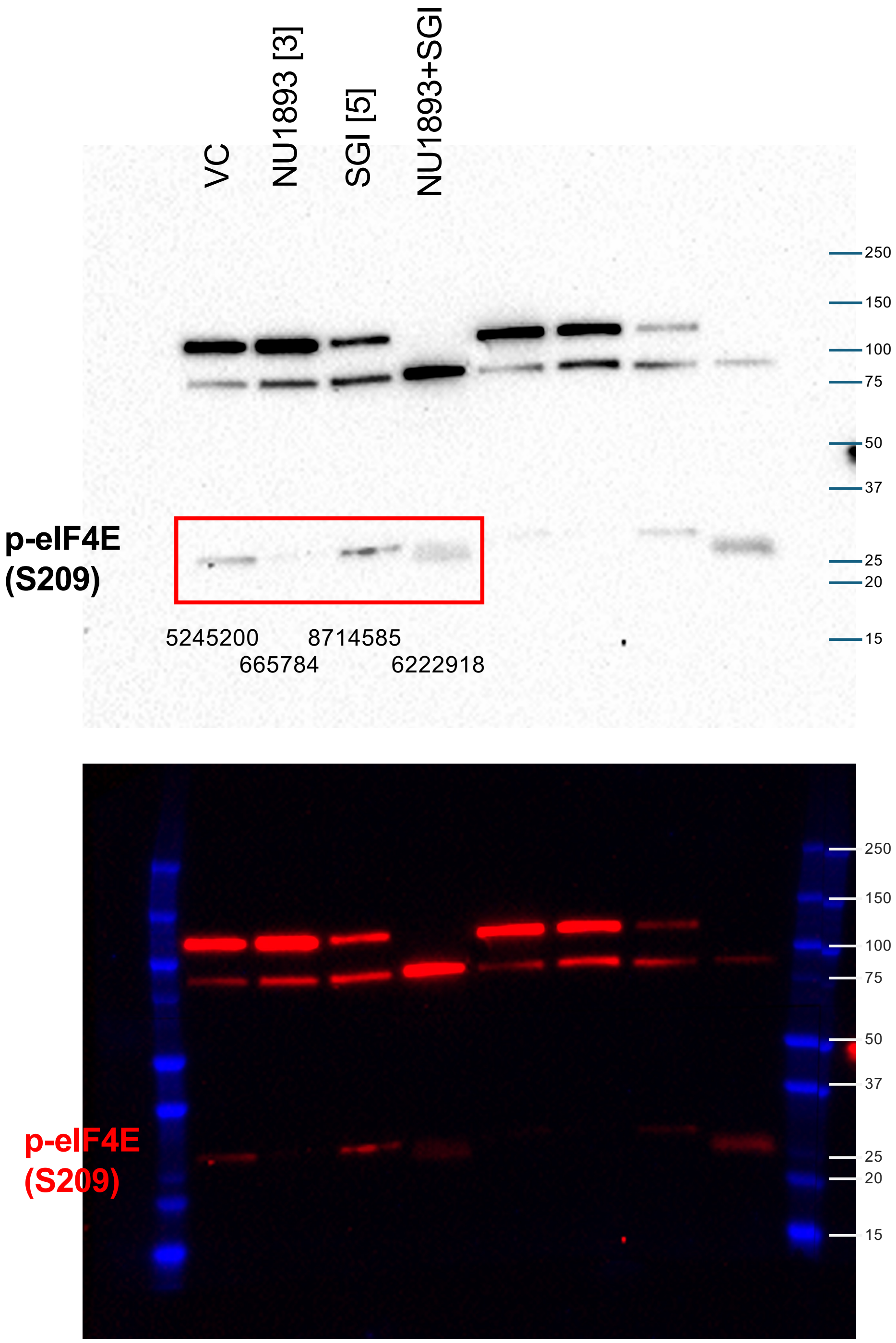

Figure S5C. Uncropped immunoblots related to **Figure 7A right panels.** Immunoblot for p-eIF4E (S209) of single chemiluminescence channel (upper) and multichannel with MW marker (lower). Immunoblot images were acquired using a ChemiDoc imaging system (Bio-Rad), and densitometric analysis was performed with Image Lab software (Bio-Rad). Band intensities were quantified using the "Adjusted Volume (Int)" values generated by the software, which are indicated below the respective bands.

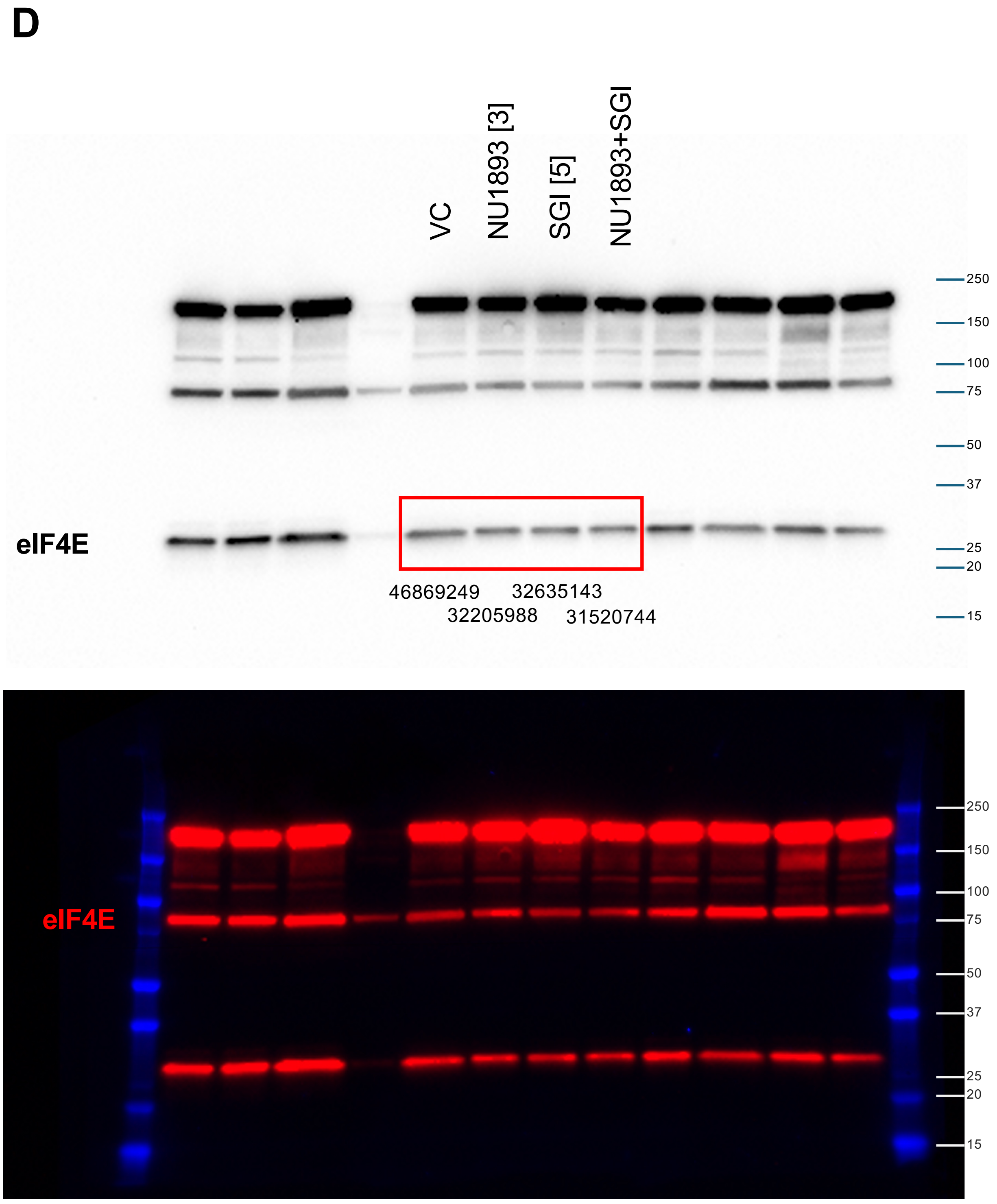

Figure S5D. Uncropped immunoblots related to **Figure 7A right panels.** Immunoblot for eIF4E of single chemiluminescence channel (upper) and multichannel with MW marker (lower). Immunoblot images were acquired using a ChemiDoc imaging system (Bio-Rad), and densitometric analysis was performed with Image Lab software (Bio-Rad). Band intensities were quantified using the "Adjusted Volume (Int)" values generated by the software, which are indicated below the respective bands.

E

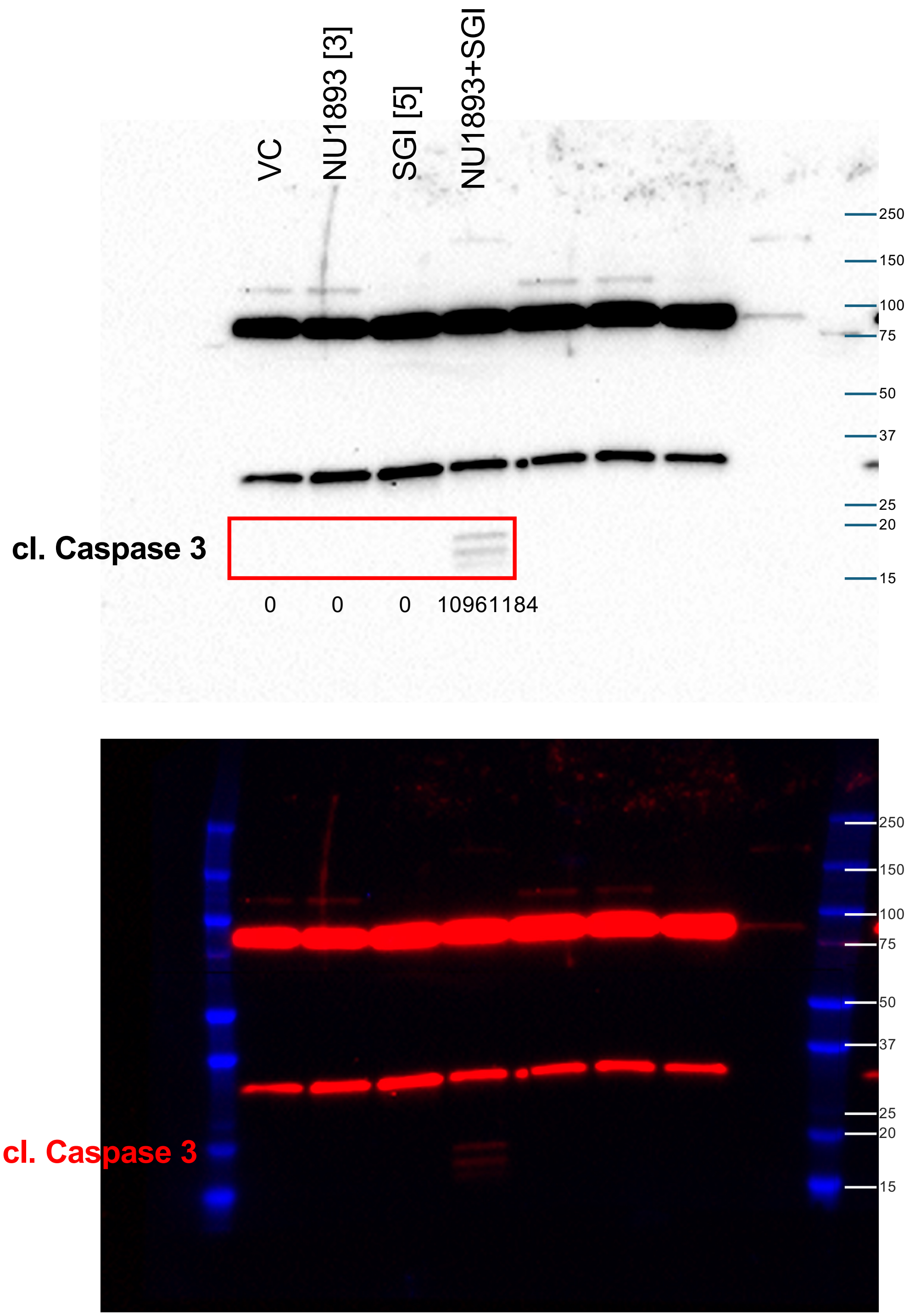

Figure S5E. Uncropped immunoblots related to **Figure 7A right panels.** Immunoblot for cleaved Caspase 3 of single chemiluminescence channel (upper) and multichannel with MW marker (lower). Immunoblot images were acquired using a ChemiDoc imaging system (Bio-Rad), and densitometric analysis was performed with Image Lab software (Bio-Rad). Band intensities were quantified using the "Adjusted Volume (Int)" values generated by the software, which are indicated below the respective bands.

F

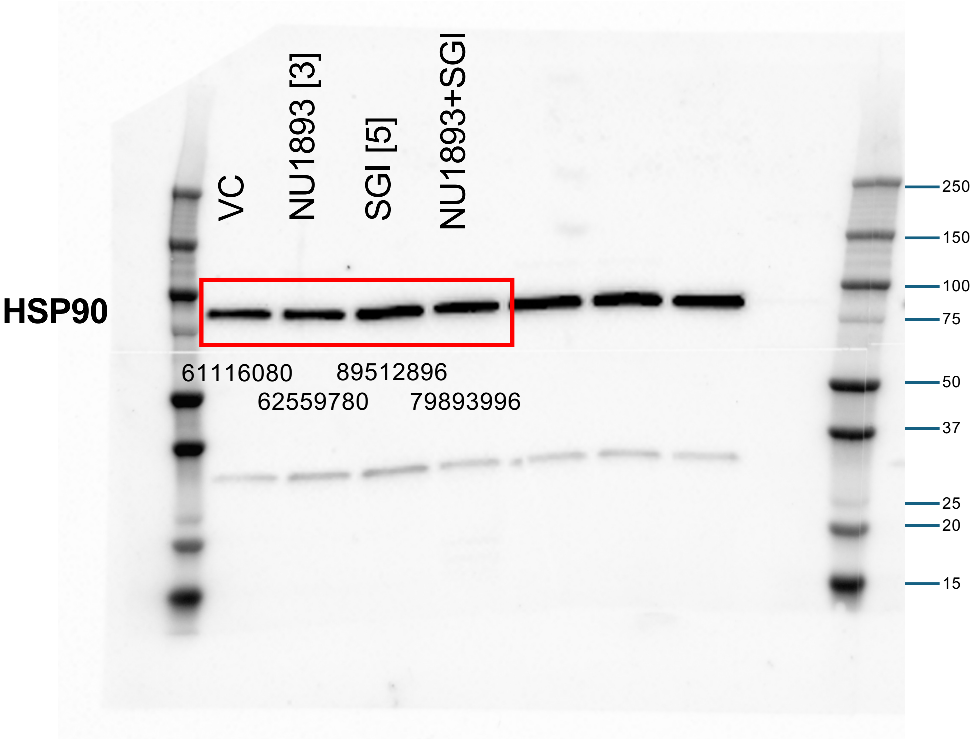

Figure S5F. Uncropped immunoblots related to **Figure 7A right panels.** Immunoblot for HSP90. Immunoblot images were acquired using a ChemiDoc imaging system (Bio-Rad), and densitometric analysis was performed with Image Lab software (Bio-Rad). Band intensities were quantified using the "Adjusted Volume (Int)" values generated by the software, which are indicated below the respective bands.

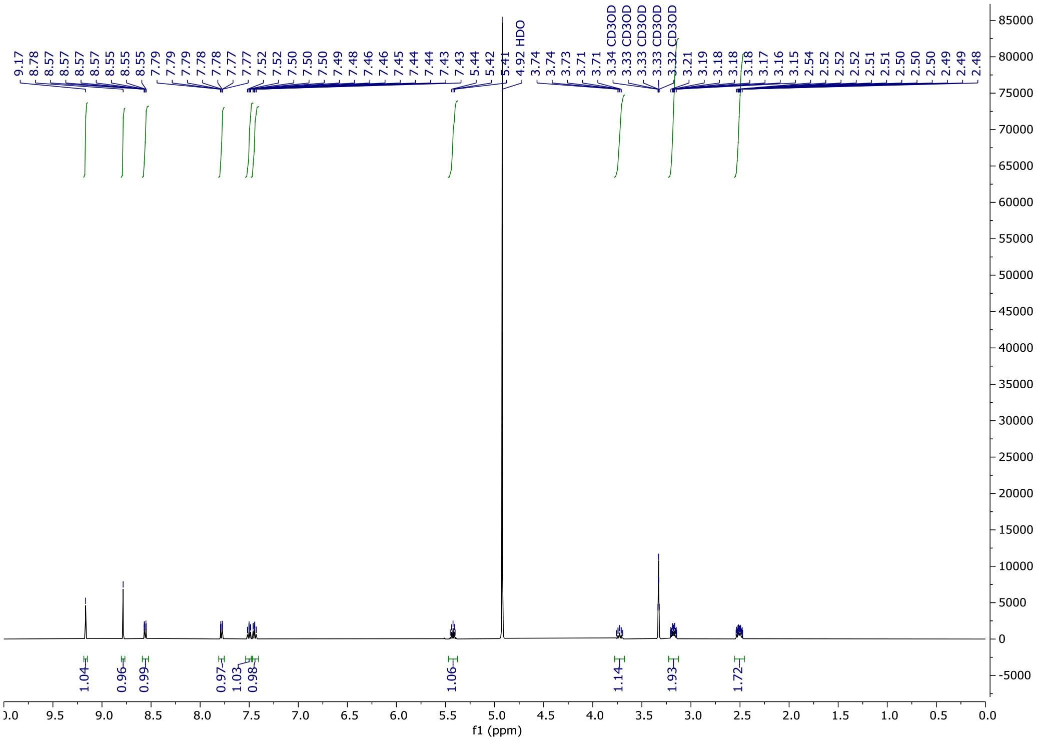

^{1}H NMR (CD_3OD) of NU1893

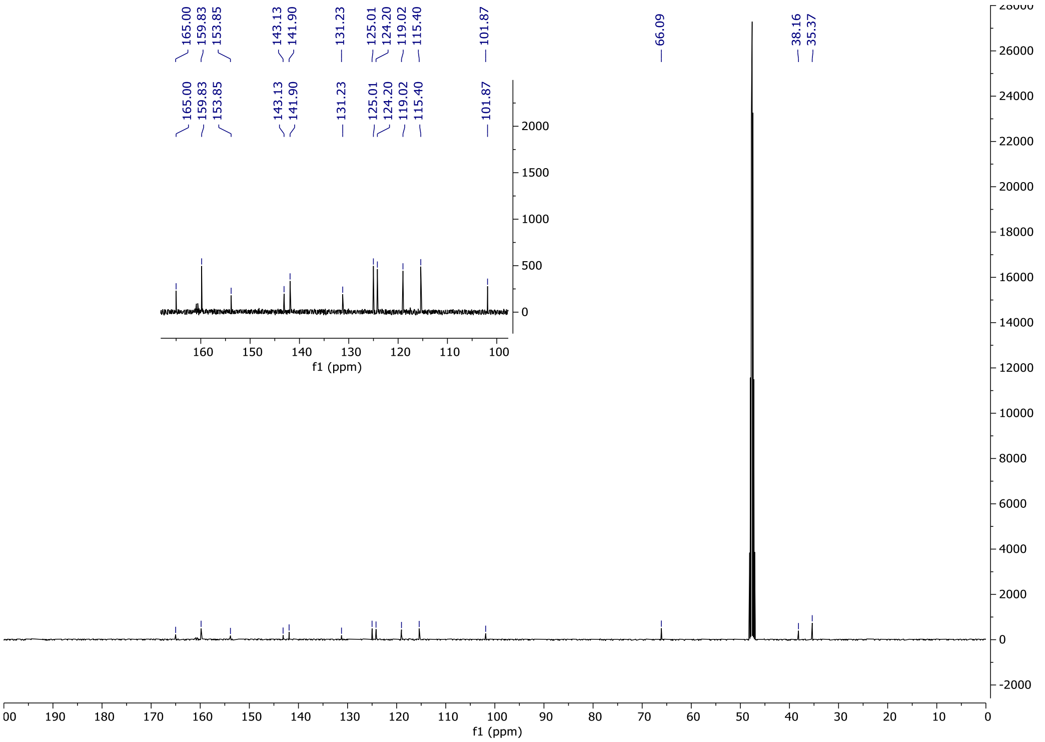

^{13}C NMR (CD_3OD) of NU1893

Figure S6. NMR spectra for NU1893. ^{1}H (top) and ^{13}C (bottom) NMR spectra in CD_3OD. In the ^{13}C NMR spectrum, a zoomed in section for aromatic peaks is shown for clarity.
